# Pyrimethamine Elicits Antitumor Effects on Prostate Cancer by Inhibiting the p38-NF-κB Pathway

**DOI:** 10.3389/fphar.2020.00758

**Published:** 2020-05-25

**Authors:** Xumin Zhou, Jinming Zhang, Xiaoping Hu, Peiqing He, Jianyu Guo, Jun Li, Tian Lan, Jumei Liu, Lilan Peng, Hua Li

**Affiliations:** ^1^Department of Pathogen Biology and Experimental Teaching Center of Preventive Medicine, Guangdong Provincial Key Laboratory of Tropical Disease, School of Public Health, Southern Medical University, Guangzhou, China; ^2^Department of Urology, Zhujiang Hospital, Southern Medical University, Guangzhou, China; ^3^Department of Respiration, Nanfang Hospital, Southern Medical University, Guangzhou, China; ^4^Department of Pharmacy, Affiliated Tumor Hospital, Guangzhou Medical University, Guangzhou, China

**Keywords:** prostate cancer, pyrimethamine, antitumor, p38 MAPK, p65/NF-κB

## Abstract

Since incurable castration-resistant prostate cancer (CRPC) inevitably develops following treatment with androgen deprivation therapy, there is an urgent need to devise new therapeutic strategies to treat this cancer. Pyrimethamine, an FDA-approved antimalarial drug, is known to exert an antitumor activity in various types of human cancer cells. However, whether pyrimethamine can inhibit prostate cancer is not well established. Hence, the present study aimed to characterize the mechanism of action of pyrimethamine on prostate cancer. We investigated the potential effect of pyrimethamine on cell proliferation, cell cycle, and apoptosis in metastatic DU145 and PC3 prostate cancer cells. We found that pyrimethamine inhibited cell proliferation, induced cell cycle arrest in the S phase, and promoted cell apoptosis of prostate cells *in vitro*; it also suppressed tumor growth in xenograft models. In addition, we observed that pyrimethamine suppressed prostate cancer growth by inhibiting the p38-NF-κB axis *in vitro* and *in vivo*. Thus, this study demonstrates that pyrimethamine is a novel p38 inhibitor that can exert antiproliferative and proapoptotic effects in prostate cancer by affecting cell cycle and intrinsic apoptotic signaling, thereby providing a novel strategy for using pyrimethamine in CRPC treatment.

## Introduction

Prostate cancer (PCa), which accounts for more than 20,000 mortalities every year, is one of the most common malignancies among male cancer patients worldwide (Edwards et al., 2014; Plata and Concepcion, 2014; Siegel et al., 2016). Recently, the incidence of PCa is rising, whereas the rate of five-year survival is declining (DeSantis et al., 2014). Although androgen deprivation therapy constitutes a standard treatment option for advanced PCa, most of the patients ultimately develop resistance to this therapy (Scher and Sawyers, 2005; Singer et al., 2008; Watson et al., 2015). Therefore, it is vital to identify new carcinogenic pathways for the treatment of castration-resistant prostate cancer (CRPC), and more efficient drugs are urgently needed because of the lack of valid chemotherapies that can provide satisfactory clinical outcomes for CRPC patients.

Pyrimethamine (2,4-diamino-5-p-chlorophenyl-6-ethyl-pyrimidine; Pyr) is a subclass of antifolate drugs that blocks the enzyme, dihydrofolate reductase, which is essential for DNA synthesis (Scher and Sawyers, 2005; Singer et al., 2008; Watson et al., 2015). It is used to treat infections caused by protozoan parasites, such as *Plasmodium falciparum* and *Toxoplasma gondii* (Masur et al., 2000; Mockenhaupt et al., 2001; Hill and Dubey, 2002). In addition to its antimalarial effects, several studies have reported that pyrimethamine might be beneficial in the treatment of different types of tumors, including lung cancer (Lin et al., 2018), melanoma (Giammarioli et al., 2008; Tommasino et al., 2016), breast cancer (Khan et al., 2018), and acute myeloid leukemia (Sharma et al., 2016). It has been suggested that the mechanism underlying this activity involves the induction of cathepsin B-dependent and caspase-dependent apoptotic pathways, inhibition of STAT3, activation of the Caspase8/9, and cell cycle arrest in S-phase. However, the specific roles of pyrimethamine and its mechanism of action in the treatment of CRPC remain unclear. A previous study has reported that the administration of imidazopyrimidine p38 MAPK inhibitor combined with pyrimethamine resulted in improved survival of mice infected with *Toxoplasma gondii* (Wei et al., 2007). As they have similar chemical groups, we hypothesized whether pyrimethamine elicits an antitumor effect via inhibiting p38 MAPK in prostate cancer (PCa). To elucidate this, we investigated the effects of pyrimethamine on tumorigenesis and progression of CRPC.

## Materials and Methods

### Reagents

Pyrimethamine (purchased from Sigma, ShangHai, China) was dissolved in dimethyl sulfoxide (DMSO) to a final concentration of 100 mmol/L. The p38 MAPK inhibitor, SB202190 (FHPI), was procured from Selleck Chemicals (Houston, USA), and the recombinant tumor necrosis factor alpha (TNF-α) was obtained from Huaxia Ocean Technology (Beijing, China). In all the experiments, the final DMSO concentration was <0.1%, and DMSO alone had no noticeable effects on the cultured cells.

### Cell Culture

Human CRPC cell lines, DU145, and PC3, were purchased from the American Type Culture Collection (ATCC) and were tested and authenticated by karyotyping analysis on 10th December, 2017. Cells were cultured in RPMI-1640 medium (Gibco, China) supplemented with 10% fetal bovine serum (FBS) (Gibco, China) and incubated at 37°C in an atmosphere of 5% CO_2_.

### Cell Viability Analysis

2 × 10^3^ DU145 and PC3 cells were seeded in 96-well plates and cultured with increasing concentrations of pyrimethamine (32 µM to 100 µM). Cell viability was assessed using the Cell Counting Kit-8 (CCK-8) (TongRen, China) according to the manufacturer's instructions and as previously described (Zhou et al., 2017). In brief, 10 µl CCK-8 solution was added into each plate, and then the optical density (OD) of the cell suspension was measured at an absorbance 450 nm after 2 h of incubation at 37°C using a microplate reader (Multiskan™ FC, Thermo Scientific). Three duplicate wells were set up for each cell group.

### Colony Formation Assays

To study the effect of pyrimethamine on the ability of DU145 and PC3 cells to form colonies, 500 cells were seeded into 6-well plates and incubated with 0, 32, and 100 µM pyrimethamine for 10 days. After 10 days, cells were washed thrice with cold PBS, and then fixed with 4% paraformaldehyde for 40 min. The colonies were stained with hematoxylin for 20 min and then counted using a microscope (Multiskan™ FC, Thermo Scientific).

### Cell-Cycle Analysis

1 × 10^6^ DU145 and PC3 cells were seeded in 6-well plates and treated with 32 µM and 100 µM of pyrimethamine for 24 h and then harvested and fixed with 70% ice-cold ethanol overnight at 4°C. Next day, after washing twice with PBS, the cells were suspended in PBS and incubated with 20 µl Rnase A and 5 µl propidium iodide (PI) for 30 min at 37°C. The cell cycle phases were analyzed by flow cytometry using a BD FACSCalibur system.

### EdU Proliferation Assay

To assess cell proliferation, 2 × 10^3^ DU145 and PC3 cells were seeded in 96-well plates and were incubated under 5% CO_2_ at 37°C in a complete medium. Next day, the cells were treated with increasing concentrations of pyrimethamine (32 µM to 100 µM). After incubating the cells for 24 h, cell proliferation was measured by the incorporation of EdU (5-ethynyl-2′-deoxyuridine) using the EdU Cell Proliferation Assay Kit (KaiJi, NanJing, China). The treated cells were incubated with 50 µM EdU for 6 h at room temperature before fixation, permeabilization, and EdU staining, which were performed according to the manufacturer's instructions. The cell nuclei were stained with DAPI (KaiJi, NanJing, China) at a concentration of 1 µg/ml for 15 min. The proportion of cells in which EdU was incorporated was detected with fluorescence microscopy (Multiskan™ FC, Thermo Scientific).

### Cell Apoptosis Analysis

To analyze whether pyrimethamine affects apoptosis, 1 × 10^6^ DU145 and PC3 cells were exposed to 32 µM and 100 µM of pyrimethamine for 24 h and then harvested and incubated with 5 µl FITC conjugated annexin V and 5 µl propidium iodide (PI) according to the manufacturer's protocol. The percentage of apoptotic cells in the treated cells was measured by flow cytometry system (BD FACSCalibur).

### Tumor Growth in Xenografts

Sixteen nude mice (4–5-week-old) were acquired from the Experimental Animal Center of Southern Medical University (Guangzhou, China) and housed under specific pathogen-free conditions. PC3 cells (6 × 10^6^) were implanted subcutaneously into the right armpit region of each nude mouse. When the tumor volume reached approximately 120 mm^3^, the mice were randomly divided into two groups and were orally administered either pyrimethamine (15 mg/kg) or vehicle control, once daily. After 30 days of treatment, all the mice were sacrificed, and tumor weights and volumes were immediately measured. Tumor volumes were calculated according to the following formula: V = [length (mm) × width^2^ (mm)] / 2. All operations were performed in accordance with the guidelines laid down in the “NIH Guide for the Care and Use of Laboratory Animals (20) (Revised 2011)” and were approved by the research ethics committee of the Southern Medical University (Guangzhou, China).

### Immunohistochemical Analysis and Evaluation Criteria

For immunohistochemistry (IHC), the tissues were deparaffinized, and later rehydrated by incubation with xylene and ethanol; the endogenous antigens were restored by autoclaving for 15 min in a citric-acid buffer (10 mM citrate buffer, pH 8.0). Endogenous peroxidase activity was blocked with 3% hydrogen peroxide, following which the slides were incubated overnight with anti-Ki-67 and anti-p38 primary antibodies at 4 °C, and subsequently with HRP-conjugated secondary antibody at 37°C for 30 min. Next, the signals were detected with DAB and nuclei were counterstained with hematoxylin. All the antibodies were obtained from Abcam and diluted in the ratio of 1:100. Intensity of the stained cells was scored as follows: 0—no staining (−), 1—weak staining (+), 2—moderate staining (++), and 3—strong staining (+++). The percentage of stained cells was divided into five classes: 0 for negative cells, 1 for 1–25%, 2 for 25–50%, 3 for 50–75%, and 4 for >75%. Total scores of the stained cells ranged from 0 to 12. All the sections were defined as having low expression (0 to 7) or high expression (8 or greater) using a semiquantitative score. Assessment of the slides was performed by two pathologists.

### Real-Time RT-PCR and Western Blot Analysis

Total RNA was extracted from the cultured cells and human tissues using TRizol reagent (TAKARA, China), and a first-strand cDNA was synthesized with the PrimeScript RT Reagent Kit (TAKARA, China) according to the manufacturer's protocol. Next, reverse transcription was performed with the SuperScript First-Strand Synthesis System (Invitrogen, Carlsbad, CA) according to the manufacturer's recommendations. Real-time PCR (RT-PCR) was performed using the cDNA and SYBR-Green II (TAKARA, China) system. The relative levels of mRNAs in cells and tissues were normalized to the housekeeping gene, GAPDH and calculated using the equation 2^−△△CT^. Primers were designed using Primer Express software, and their sequences were shown in **Table 1**.

**Table 1:** Primer sequences for qRT-PCR.

Primers	Sequences 5′→3′
Human P38-F	AAGGAAGGAGGCAGACTGATGG
Human P38-R	CTGTGGATGGTGAGGATTTGAAC
Human P65-F	CCTGGTCCCGTGAAATACACCT
Human P65-R	ATCCCATCTTTGACAATCGTGC
Human GAPDH-F	AGAAGGCTGGGGCTCATTTG
Human GAPDH-R	AGGGGCCATCCACAGTCTTC

The treated cells were washed thrice with cold PBS and lysed with RIPA buffer (KaiJi, China) containing protease and phosphatase inhibitors. The concentration of proteins was detected by the bicinchoninic acid method. For western blotting, proteins were separated using 10% polyacrylamide gel electrophoresis, which were subsequently transferred onto a 0.22 μm pore-size PVDF membrane (the PVDF membrane was activated with methanol). After blocking with 5% skim milk, the membrane was incubated with the appropriate primary antibodies at 4°C overnight. Next, the membrane was washed five times for 6 min, followed by incubation with HRP-conjugated secondary antibody for 2 h at room temperature. Finally, the protein blots were visualized using ECL substrate reagents.

### Statistical Analysis

All data are shown as the mean ± S.D of at least three independent experiments. Data were analyzed by Student's t-test and one- or two-way ANOVA, as appropriate. All statistical analyses were performed using the SPSS 20.0 software. Statistical signiﬁcance of differences between groups is denoted by asterisks (*P < 0.05; **P < 0.01; ***P < 0.001).

## Results

### Pyrimethamine Inhibited Cell Proliferation in Human CRPC Cells

**Figure 1:**
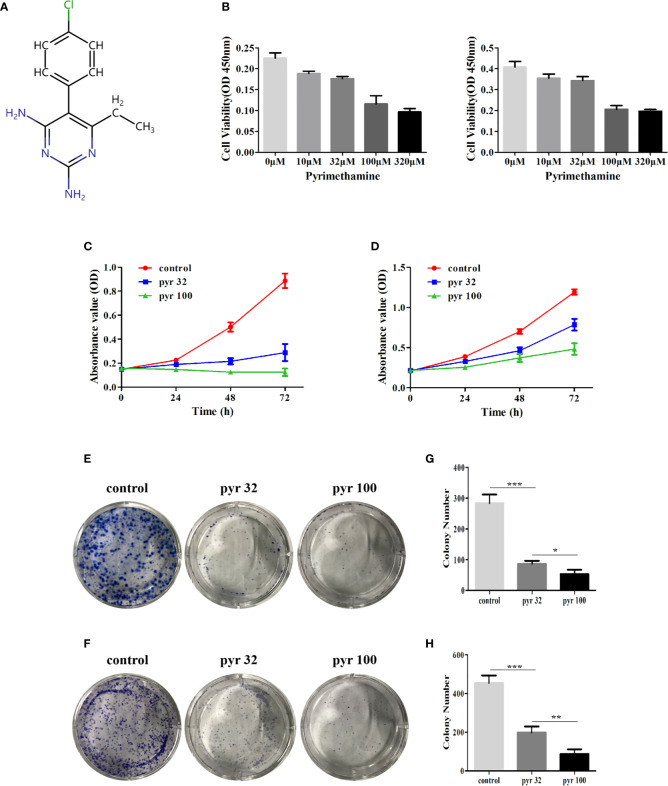
Pyrimethamine inhibits the cell proliferation in human CRPC cells. **(A)** Chemical structure of pyrimethamine. **(B)** The changing 50% inhibitory concentrations of pyrimethamine fitting in DU145 (left) and PC3 (right) cells. **(C, D)** Pyrimethamine inhibits the proliferation of DU145 **(C)** and PC3 **(D)** cells as detected by CCK-8 assays. **(E, F)** Representative images of the DU145 and PC3 cell colonies after treatment of 0, 32, and 100 μM pyrimethamine were shown. **(G, H)** Mean colony numbers ± SD were shown. Each bar represents the mean ± SD of three independent experiments. *P < 0.05; **P < *0.01*; ***P < 0.001.

The chemical structure and the ADMET data of pyrimethamine are shown in **Figure 1A** and **Table 2**, respectively. To explore the possible cytotoxic effect of pyrimethamine in human CRPC cell growth, DU145 and PC3 cells were exposed to the increasing concentrations of pyrimethamine (from 0 to 320 μM) for 24 h. The changing 50% inhibitory concentrations of pyrimethamine fitting in DU145 (left) and PC3 (right) cells were about 80 and 100 μM (**Figure 1B**). A CCK-8 assay demonstrated that pyrimethamine significantly decreased the viability of CRPC cells (**Figures 1C, D**). In addition, a colony formation assay revealed that pyrimethamine inhibited the clonality of DU145 (*P* < 0.001) and PC3 cells (*P* < 0.001) (**Figures 1E–H**).

**Table 2:** The ADMET data is predicted using admetSAR, a free tool for evaluating chemical ADMET properties. This three line watch is still short of a bottom line.

Property	Value	Probability
Human intestinal absorption	+	0.9973
Blood brain barrier	+	0.9383
Renal organic cation transporter	Noninhibitor	0.7451
Ames test	Non-AMES toxic	0.9133
Carcinogenicity	Noncarcinogens	0.8016
Biodegradation	Not readily biodegradable	1.0
P-glycoprotein substrate	Nonsubstrate	0.5822

### Pyrimethamine Arrested the Cell Cycle at S Phase

**Figure 2:**
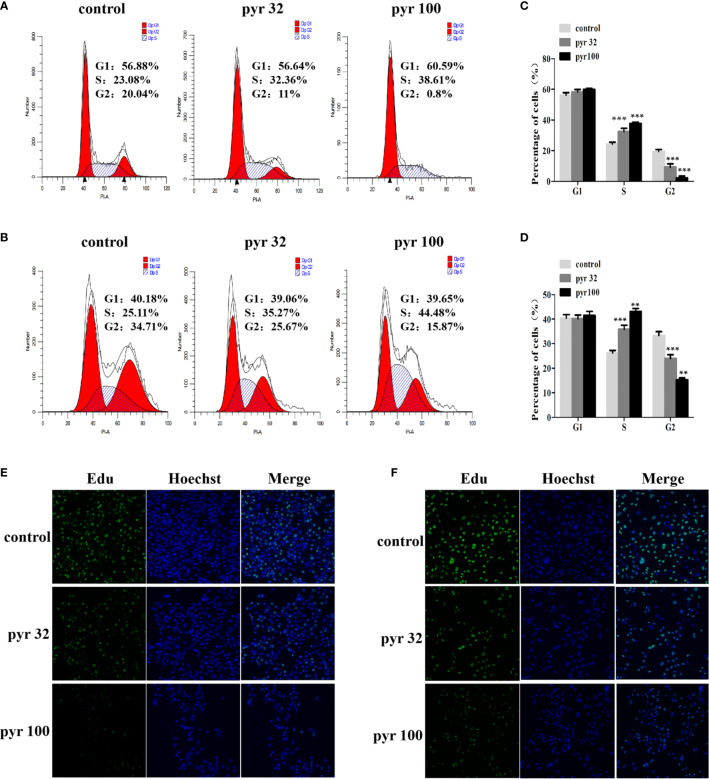
Pyrimethamine arrests the cell cycle at S phase. **(A, B)** Analyzing the cell cycle arrest after treating with 0, 32, and 100 μM pyrimethamine in DU145 and PC3 cell lines by flow cytometry. **(C, D)** Mean percentage of cells ± SD was shown (DU145 and PC3). **P < *0.01*; ***P < 0.001. **(E, F)** Pyrimethamine represses the DNA synthesis of DU145 **(E)** and PC3 **(F)** cells as detected by EdU proliferation assay.

The abovementioned results showed that pyrimethamine suppressed the proliferation of DU145 and PC3 cells. To investigate the effect of pyrimethamine on cell cycle, which plays a major role in cell proliferation, we carried out flow cytometry analysis. By analyzing the cell cycle distribution of DU145 (**Figures 2A, C**) and PC3 (**Figures 2B, D**), we found that the percentage of G2 phase cells was decreased from 33.32 ± 1.56, 19.41 ± 1.33 to 24.01 ± 1.50, 15.31 ± 0.71 and 9.25 ± 2.19, 2.20 ± 1.21 in DU145 and PC3 cells, respectively, with increasing concentrations of pyrimethamine (*P* < 0.001). In contrast, the percentage of S phase cells was increased from 26.23 ± 1.53, 24.37 ± 1.17 to 35.82 ± 1.66, 43.15 ± 1.20 and 32.43 ± 2.20, 37.78 ± 0.78, in DU145 and PC3 cells, respectively (*P* < 0.001). It is widely known that synthesis of DNA mainly occurs in the S phase; therefore, we examined its change in DU145 and PC3 cells after treating with pyrimethamine using the EdU assay (**Figures 2E, F**). We found that pyrimethamine significantly reduced the synthesis of DNA. All these data revealed that pyrimethamine inhibited the proliferation of DU145 and PC3 cells by inducing cell cycle arrest in the S phase.

### Pyrimethamine Affected Tumorigenesis of PC3 Cells *In Vivo*

**Figure 3:**
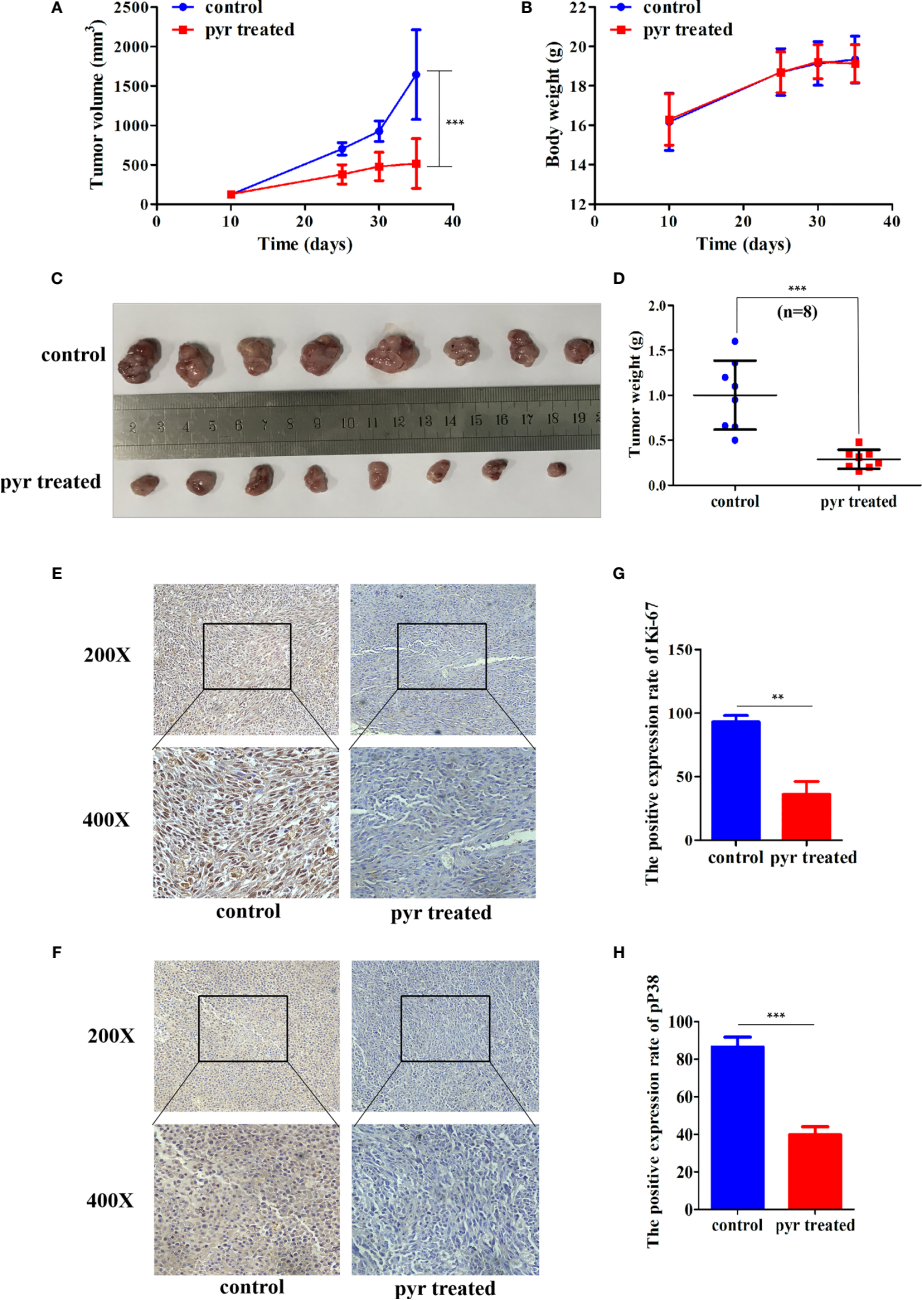
Pyrimethamine affects tumorigenesis of PC3 cells *in vivo*. **(A)** Tumor volumes were measured on the indicated days and drew into the tumor growth curves. **(B)** The body weight of each mouse was monitored during the experiment. **(C)** Image of tumors dissected from 16 nude mice (n = 8 for each group). 6 × 10^6^ PC3 cells were implanted subcutaneously into the right armpit regions of each nude mouse. After injection 10 days (~120 mm^3^), mice were randomly distributed into two groups, pyrimethamine treated group (15 mg/kg/d) and the control group. **(D)** The tumors' weight were weighed and presented by scatter plot. **(E, F)** Representative immunohistochemistry staining images of Ki-67 and P38 from tumor tissues in each group. **(G, H)** Mean positive rates ± SD were shown (n = 8). Data represent the mean ± SD of three independent experiments. **P < *0.01*; ***P < 0.001 (*vs.* Control).

As pyrimethamine expressed antiproliferation activity in CRPC cells *in vitro*, we further explored whether it could inhibit tumorigenicity *in vivo*. To clarify the effect of pyrimethamine on tumor growth, we carried out a xenograft tumor assay in nude mice. PC3 cells (6 × 10^6^) were implanted subcutaneously into the right armpit region of each nude mouse. When the tumor volume reached approximately 120 mm^3^, the mice were randomly divided into two groups and were orally administered either pyrimethamine (15 mg/kg) or vehicle control, once daily. After 30 days of treatment, we found that tumor growth was suppressed in the pyrimethamine-treated group compared to that in the control group (*P* < 0.001) (**Figures 3A, C**). However, the body weights of mice showed no significant difference between the two groups (*P* >0.05). Moreover, the behavior, overall activity, and feeding pattern of mice did not display any obvious changes. To further identify the inhibitory effect of pyrimethamine on tumor growth, we performed immunohistochemical analysis to detect the expression of Ki-67 and pp38 in paraffin-embedded mice tumors. We found that the expression of Ki-67 and pp38 in the pyrimethamine-treated group was lower than that in the control group. The above results suggested that pyrimethamine significantly suppressed tumor growth in the xenograft model of nude mice by exhibiting favorable toxicological profiles.

### Pyrimethamine Promoted the Apoptosis of CRPC Cell Lines

**Figure 4:**
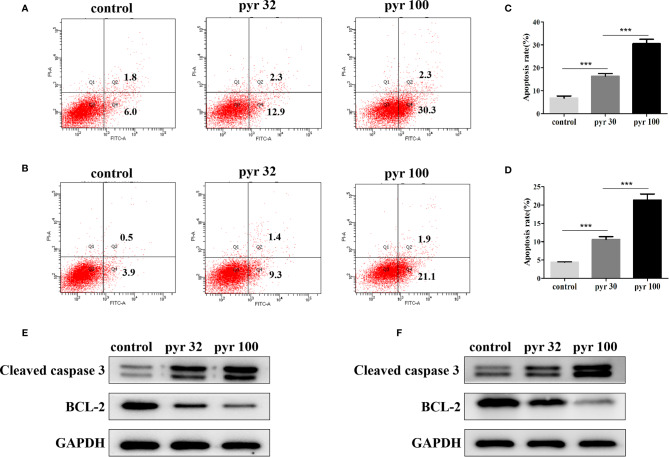
Pyrimethamine promotes the apoptosis of CRPC cell lines. **(A, B)** Pyrimethamine promotes the apoptosis of DU145 **(A)** and PC3 **(B)** cells, which were analyzed by flow cytometric using Annexin V-FITC/PI staining. **(C, D)** Statistical results were represented as mean ± SD of three independent experiments. ***P < 0.001. **(E, F)** The expression of Bcl-2, caspase-3 was identified by western blotting after treating with 0, 32, and 100 μM pyrimethamine for 24 h (DU145 and PC3). GAPDH was used as a loading control.

Furthermore, we examined the involvement of pyrimethamine in CRPC cell apoptosis after treatment with increasing concentrations of pyrimethamine (from 32 to 100 μM) by flow cytometry analysis. Our results indicated that the apoptotic rates in DU145 and PC3 cells in the pyrimethamine-treated group were markedly higher than those in the control group (*P* < 0.001) (**Figures 4A–D**). To the best of our knowledge, the intrinsic apoptotic pathway plays an important role in cell apoptosis. Therefore, we also measured the expression of BCL-2 and cleaved caspase 3 after treating the cells with pyrimethamine. The results revealed that pyrimethamine downregulated the expression of BCL-2 but upregulated the expression of cleaved caspase 3 (**Figures 4E, F**). All these results demonstrated that pyrimethamine could promote apoptosis in the DU145 and PC3 cells.

### Pyrimethamine Inhibited Proliferation and Promoted Apoptosis in CRPC Cells by Inhibiting p38 MAPK

**Figure 5:**
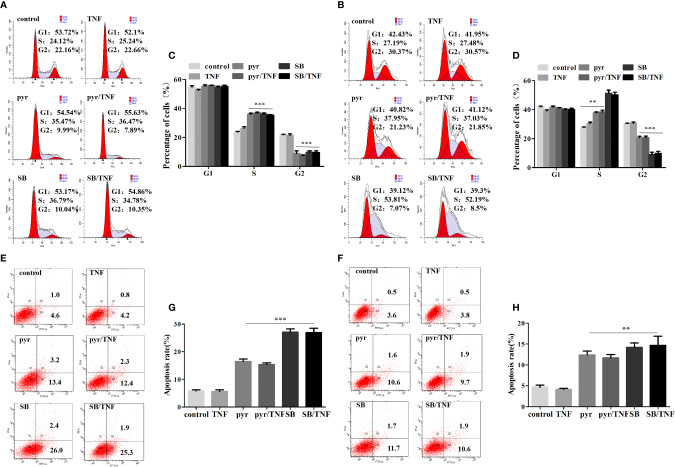
Pyrimethamine inhibits proliferation and promotes apoptosis in CRPC cells through the inhibition of p38 MAPK. **(A, B)** DU145 and PC3 cells were treated with pyrimethamine or SB202190 (10 μM) for 24 h and then incubated with TNF-a (10 ng/ml) for 24 h, and quantification of percentages in cell cycle phases was analyzed by flow cytometry. **(C, D)** Mean percentage of cells ± SD was shown (DU145 and PC3). **:P < 0.01; ***:P < 0.001. **(E, F)** The apoptosis of the treated DU145 and PC3 cells was detected by flow cytometry. **(G, H)** The graph shows the statistical results obtained from three independent experiments. **:P < 0.01; ***:P < 0.001.

Previous studies have identified imidazopyrimidine as a p38 MAPK inhibitor in treating *Toxoplasma gondii* infection (Wei et al., 2007). As imidazopyrimidine and pyrimethamine have similar chemical groups, we investigated whether pyrimethamine elicits an antitumor effect by inhibiting p38 MAPK in PCa. To this end, the cells were incubated with pyrimethamine (32 μM) or a specific p38 MAPK inhibitor, SB202190 (10 μM) and then treated for the indicated time in the absence or presence of TNF-α (10 ng/ml), which was used as a p38 MAPK inducer. Later, flow cytometry was performed to detect cell proliferation and apoptosis. Expectedly, we observed that both pyrimethamine and the p38 MAPK inhibitor produced a significant increase in the percentage of the S phase cells and number of apoptotic cells after TNF-α-treatment compared to that of the control group, which was also activated by incubation with TNF-α (**Figures 5A–D**). Interestingly, similar to the specific p38 MAPK inhibitor, the effect of pyrimethamine on cell apoptosis was not rescued by TNF-α (**Figures 5E–H**). Collectively, these results demonstrate that pyrimethamine induces apoptosis in CRPC cells by inhibiting p38 MAPK.

### Pyrimethamine Regulates the p65/NF-κB Pathway in CRPC Cells Through p38 MAPK Inhibition

**Figure 6:**
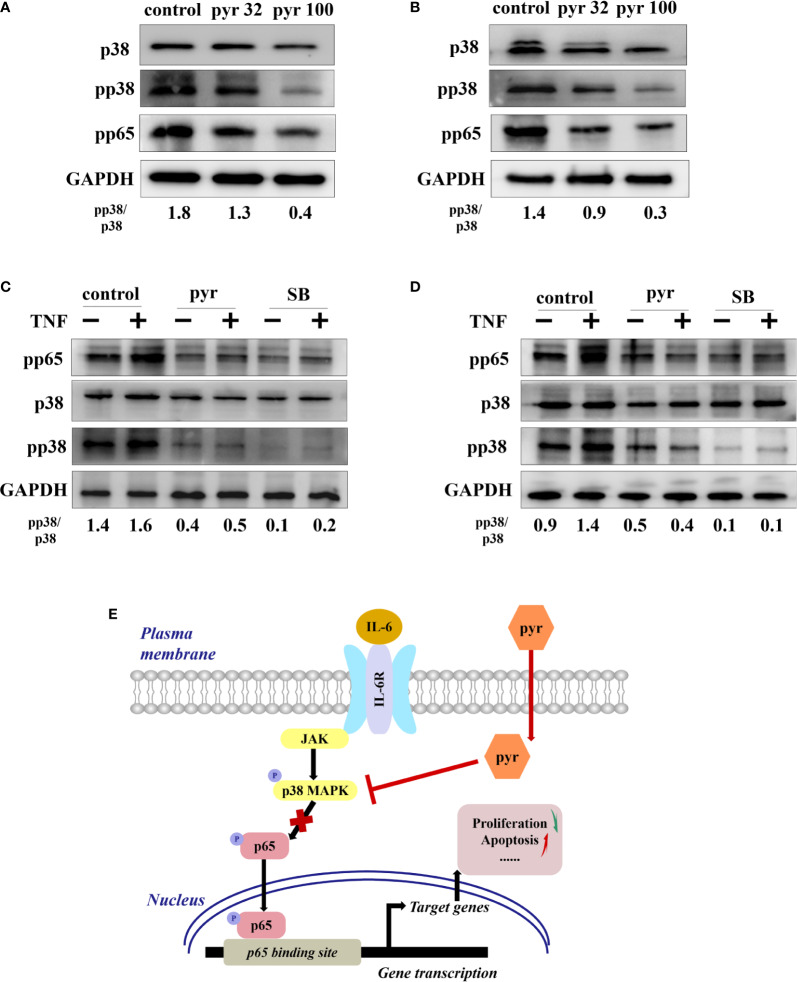
Pyrimethamine regulates the p65/NF-*k*B pathway in CRPC cells through p38 MAPK inhibition. **(A, B)** The western blot analysis of the expression of p38, pp38, and pp65 after treatment of 0, 32, and 100 μM pyrimethamine for 24 h in DU145 and PC3 cell lines. The ratio of pp38/p38 was normalized to GAPDH. **(C, D)** DU145 and PC3 cells were treated for 24 h with pyrimethamine or SB202190 (10 μM) and then treated for 6 h in the absence or presence of TNF-a (10 ng/ml). Total cell lysates were analyzed by western blot with antibodies against p38, pp38, and pP65. **(E)** A consolidated model that illustrates a plausible sequence for the mechanism by which pyrimethamine elicits antitumor effects in prostate cancer.

To further explore the mechanism through which pyrimethamine regulates the proliferation and apoptosis of CRPC cells, we examined its effect on p38 and p65, as p65/NF-*κ*B is a very important regulator of cell survival (Chopra et al., 2004; Rodriguez-Berriguete et al., 2012), which is activated by p38 MAPK in CRPC cells(Vanden et al., 1998; Baeza-Raja and Munoz-Canoves, 2004; Calleros et al., 2006; King et al., 2009; Lasa et al., 2010). Our results revealed that pyrimethamine significantly decreased the expression of pp38, pp65, and the pp38/p38 ratio in a dose-dependent manner (from 32 to 100 μM) in the CRPC cells (*P* < 0.001), without changing the total p38 level (**Figures 6A, B**). In our previous studies, we had confirmed that pyrimethamine inhibits p38 MAPK activity and that this suppression promotes apoptosis in CRPC cells; accordingly, we hypothesized whether the inhibition of p38 MAPK, achieved by either treating with pyrimethamine or SB202190 (a speciﬁc p38 MAPK inhibitor) also impaired p65/NF-*κ*B activity in these cells. Consequently, we performed experiments to detect the effect of pyrimethamine and SB202190 on NF-*κ*B activity. The results demonstrated that TNF-α signiﬁcantly increased p65/NF-*κ*B activity and treating CRPC cells with either pyrimethamine or SB202190 prevented both basal and TNF-α induced NF-*κ*B activity. These results demonstrate that pyrimethamine negatively regulates p65/NF-*κ*B activity (**Figures 6C, D**) in CRPC cells through a mechanism involving the inhibition of p38 MAPK.

## Discussion

With an improvement in the standard of living in humans, the incidence and mortality of PCa have increased. Presently, the first-line treatment of PCa is mainly androgen deprivation therapy and surgery, which is effective for hormone-dependent PCa; however, most of the patients with PCa eventually progress to the more aggressive castration resistant prostate cancer (CRPC) (Scher and Sawyers, 2005; Singer et al., 2008; Watson et al., 2015), for which there is no currently available effective treatment. Therefore, exploring new and available treatment options has always been a challenging problem to overcome in the field of urology.

Pyrimethamine, an FDA-approved antimalarial drug, interferes with the regeneration of tetrahydrofolic acid from dihydrofolate by targeting dihydrofolate reductase of the plasmodium, which is a cofactor required for DNA synthesis (Hansford, 1974; Kumpornsin et al., 2014). Consistent with this notion, we observed that pyrimethamine arrested cell cycle at the S phase. Moreover, EdU proliferation analysis revealed that pyrimethamine significantly reduced the synthesis of DNA. Pyrimethamine is a safe and efficacious antimalarial drug, which is widely used for treating infections caused by *Toxoplasma gondii* and *Plasmodium falciparum* (Masur et al., 2000; Mockenhaupt et al., 2001; Hill and Dubey, 2002). In addition to its antiprotozoal effects, pyrimethamine is also thought to possess immunomodulatory activities, including inducing apoptosis in peripheral blood lymphocytes (Gilbreath et al., 1983; Egeli, 1998; Pierdominici et al., 2005). Recent studies suggest that pyrimethamine exhibits inhibitory activity against several cancers. However, the specific roles of pyrimethamine in CRPC remain unclear. In the present study, we described that pyrimethamine exerted an antitumor activity in human CRPC cell lines. A xenograft tumor assay in nude mice indicated that pyrimethamine also possessed antitumor potential *in vivo*. Furthermore, our study demonstrated that the body weights of mice showed no significant difference between the pyrimethamine-treated and vehicle control groups, indicating favorable toxicological effects in the mice.

A previous study demonstrated that administration of imidazopyrimidine, a p38 MAPK inhibitor, improves the survival of mice infected with *Toxoplasma gondii* (Wei et al., 2007). As imidazopyrimidine and pyrimethamine possess similar chemical groups, we investigated whether pyrimethamine elicits an antitumor affect by inhibiting p38 MAPK in CRPC.

The p38 mitogen-activated protein kinase (MAPK) pathway is a key signal transduction pathway that acts to sense and adapt to external stimuli. Additionally, it controls numerous cellular functions, including proliferation, cell mobility, and apoptosis. The p38 MAPK family consists of four isoforms (p38-MAPK*α*, p38-MAPK*β*, p38-MAPK*γ*, and p38-MAPK*δ*), which share varied degrees of sequence homology and activity (Zou and Blank, 2017). Several studies have suggested that p38 MAPK is activated in human cancers and is commonly correlated with poor prognosis (Hong et al., 2015; Cao et al., 2016; Mo et al., 2016; Yuan et al., 2016; Cheng and Cao, 2017). Furthermore, small molecular inhibitors of p38 MAPK have been gradually utilized to overpower cancer cells (Campbell et al., 2014; Gilani et al., 2016; Chowchaikong et al., 2018). Interestingly, the p38 MAPK pathway plays a dual role in tumorigenesis and tumor-suppression in PCa (Tanaka et al., 2003; Xu et al., 2015; Park et al., 2017). In this study, we showed that pyrimethamine inhibited the expression of pp38, without affecting the total P38. These data partly shed light on the mechanism by which pyrimethamine exerts antitumor activity in human CRPC cells.

The nuclear factor *κ*B (NF-*κ*B) is a well-known transcription factor in the regulation of immune responses and inflammation. Moreover, growing evidence suggests that it plays a major role in the development and progression of cancer, such as proliferation, invasion, and apoptosis (Dolcet et al., 2005). The NF-*κ*B family comprises five members, namely, p50, p52, p65 (RelA), c-Rel, and RelB. Notably, it has been demonstrated that NF-*κ*B mediates the migration of and resistance to castration in PCa cells (Jin et al., 2014; Kim et al., 2017). Especially, a large cohort of European men revealed that NF-*κ*B p65 might be a prognostic biomarker in PCa (Gannon et al., 2013). Interestingly, it has been demonstrated that NF-*κ*B is activated by p38 MAPK in several cellular contexts. Our results consistently showed that TNF-α, a p38 MAPK inducer, significantly increased p65/NF-*κ*B activity, and treating CRPC cells with pyrimethamine or the p38 MAPK inhibitor prevented both basal and TNF-α induced NF-*κ*B activities. This effect was also confirmed by flow cytometry analysis in our study. These data suggest that pyrimethamine negatively regulates p65/NF-*κ*B activity in CRPC cells through a mechanism involving p38 MAPK inhibition (**Figures 6E**).

## Conclusion

Thus, our study reveals that pyrimethamine exerts antiproliferation and proapoptosis effects in CRPC cells. Furthermore, antitumor potential of pyrimethamine was also evaluated in a nude mice xenograft tumor model, *in vivo*. These data provide a strong evidence that pyrimethamine is a novel p38 inhibitor and indicate a novel application of pyrimethamine in the treatment of CRPC.

## Data Availability Statement

All datasets generated for this study are included in the article/**Supplementary Files**.

## Ethics Statement

All operations involving animals conformed to the Chinese National Institute of Health Guide for the Care and Use of Laboratory Animals, and the study was approved by the research ethics committee of Southern Medical University (Guangzhou, China) (NFYY-2016-54).

## Author Contributions

XZ and HL designed the research experiments. XZ, JZ, and XH performed the experiments. XZ, PH, JG, JL, TL, JmL, and LP collected and analyzed the data. XZ and HL prepared and edited the manuscript. All authors have given approval to the final version of the manuscript.

## Funding

This study was supported by the National Natural Science Foundation of China (81471980), Natural Science Foundation of Guangdong Province (2014A030313318).

## Conflict of Interest

The authors declare that the research was conducted in the absence of any commercial or financial relationships that could be construed as a potential conflict of interest.

## Abbreviations

CRPC, castration-resistant prostate cancer; Pca, prostate cancer; Pyr, pyrimethamine (2,4-diamino-5-p-chlorophenyl-6-ethyl-pyrimidine); MAPK, mitogen-activated protein kinase; NF-κB, nuclear factor κb; TNF-α, tumor necrosis factor alpha; EdU , 5-ethynyl-2′-deoxyuridine.
