# Supplementary material for: Pyrimethamine Elicits Antitumor Effects on Prostate Cancer by Inhibiting the p38-NF-κB Pathway
Source: Front Pharmacol. 2020 May 25;11:758. doi: 10.3389/fphar.2020.00758 (PMC7261869; doi:10.3389/fphar.2020.00758)
Supplement: Supplementary file 1 [file DataSheet_1.docx]

**
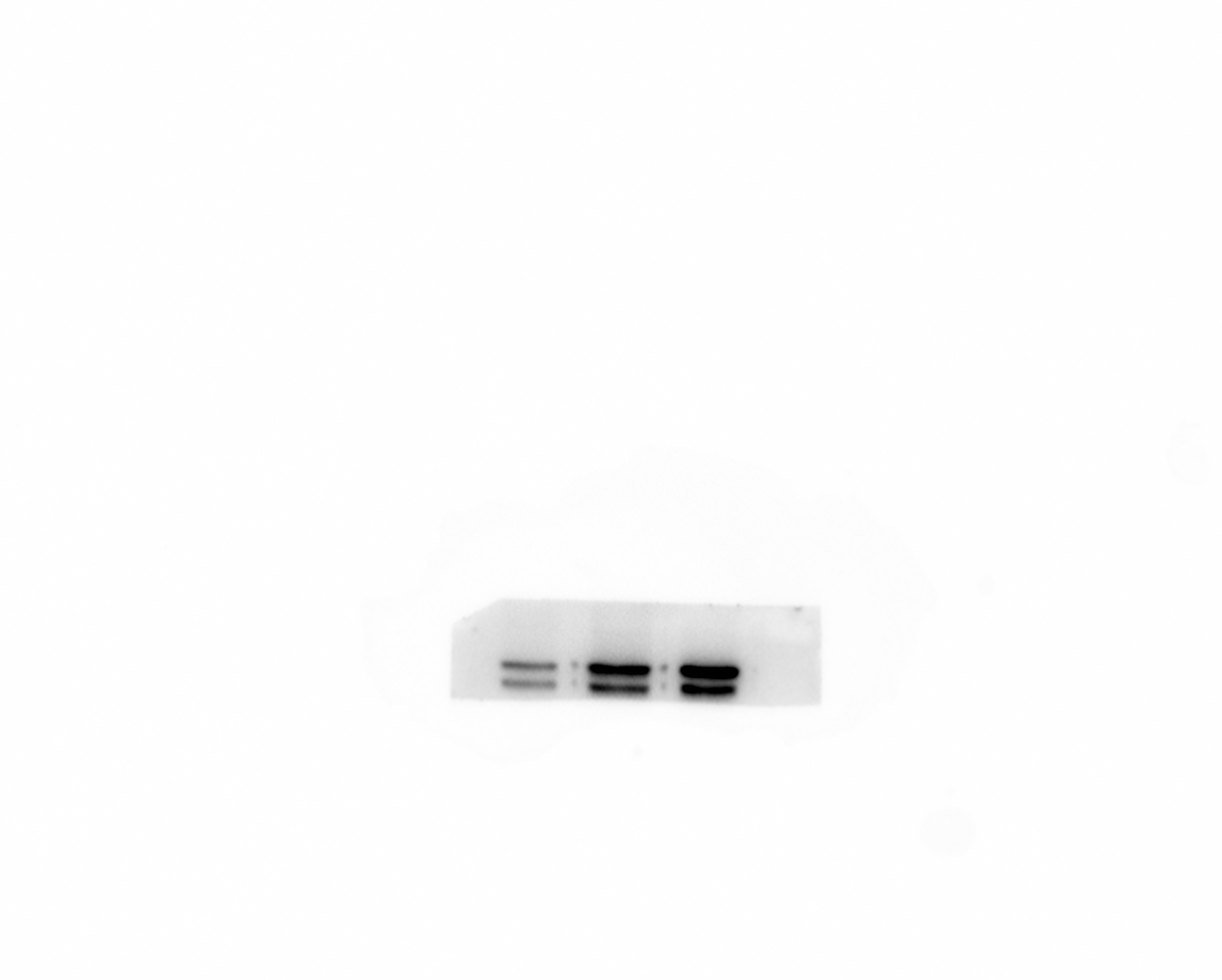
Figure 4 E**

**Cleaved caspase 3**


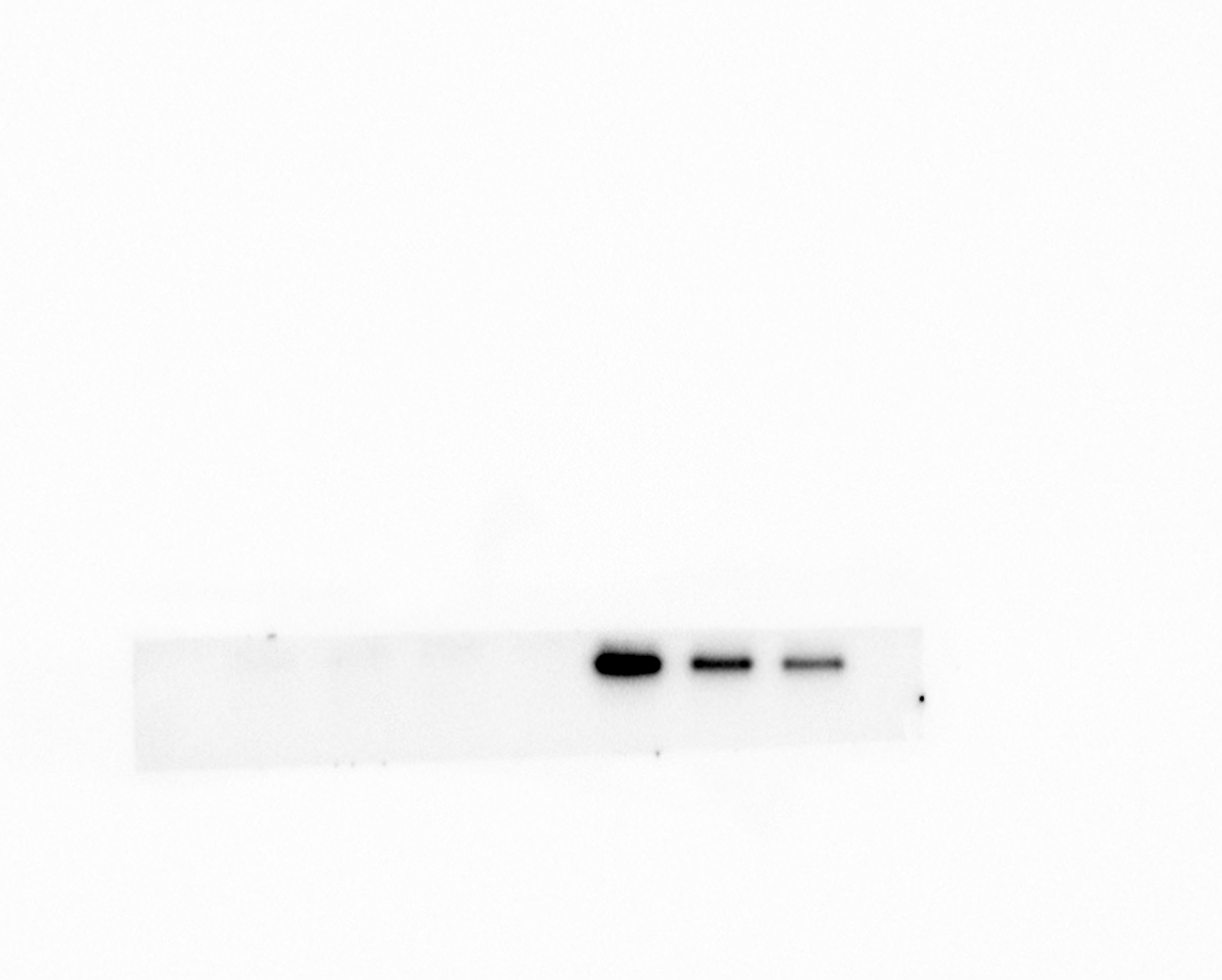


**BCL-2**


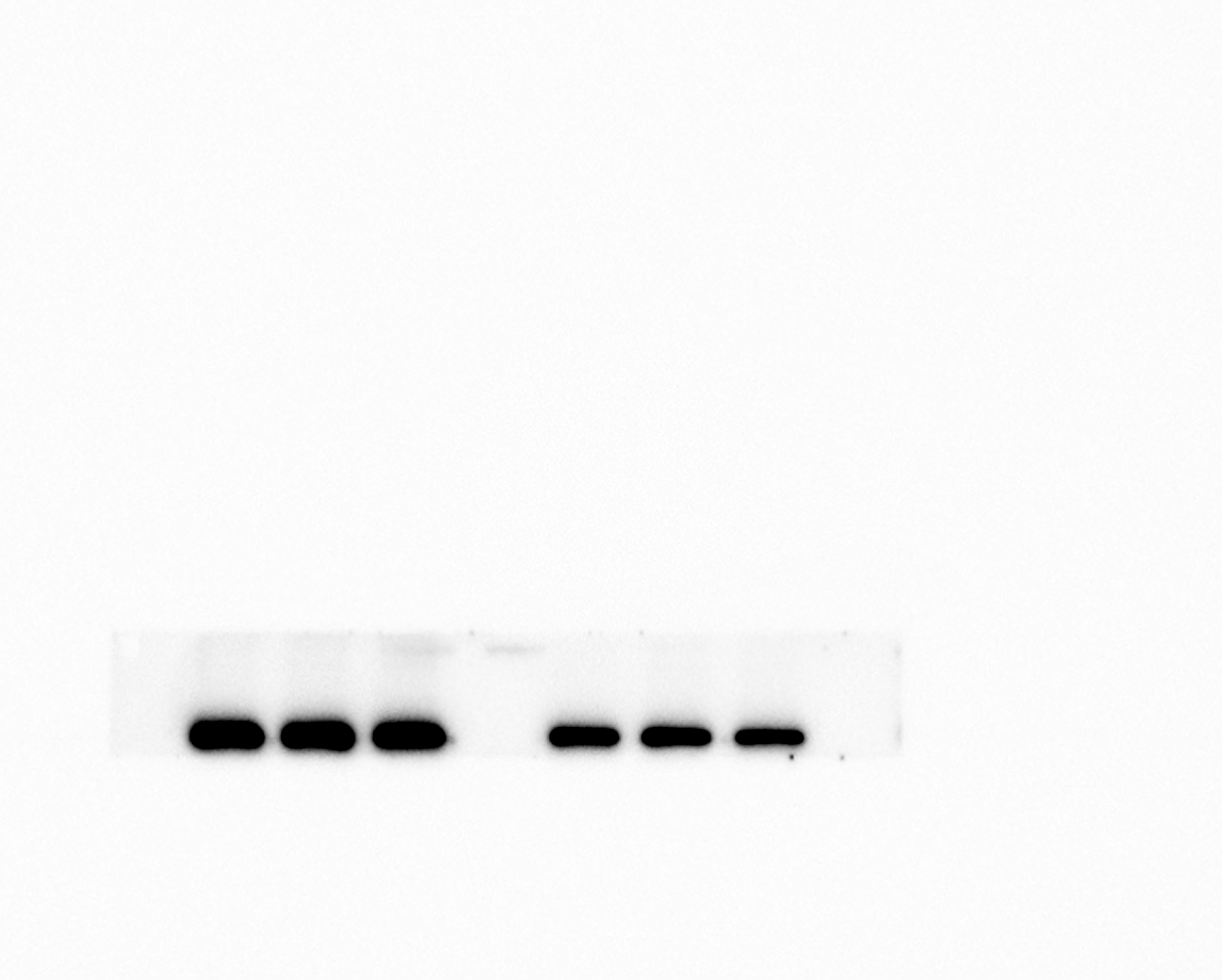


**GAPDH**

**Figure 4 F**


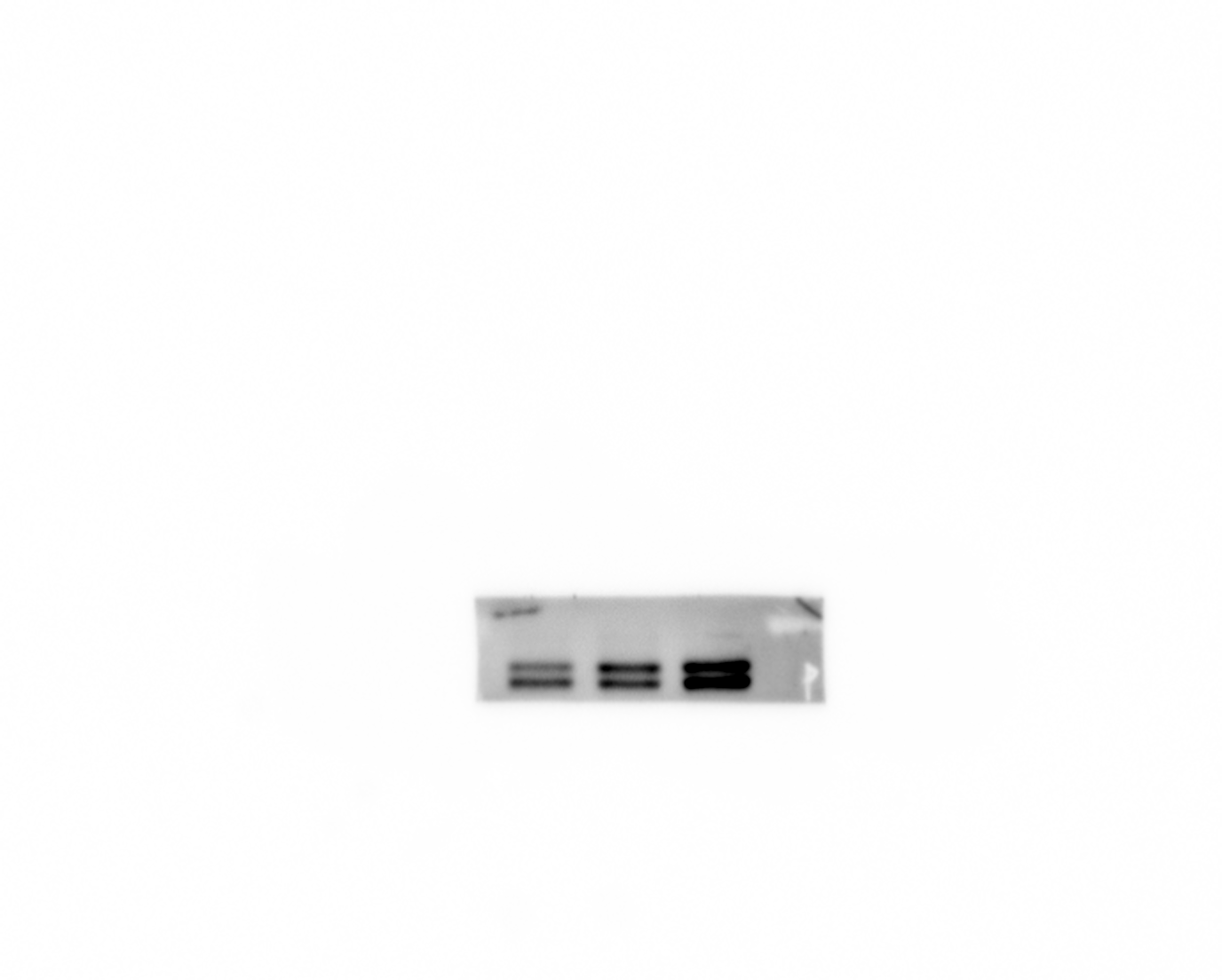


**Cleaved caspase 3**


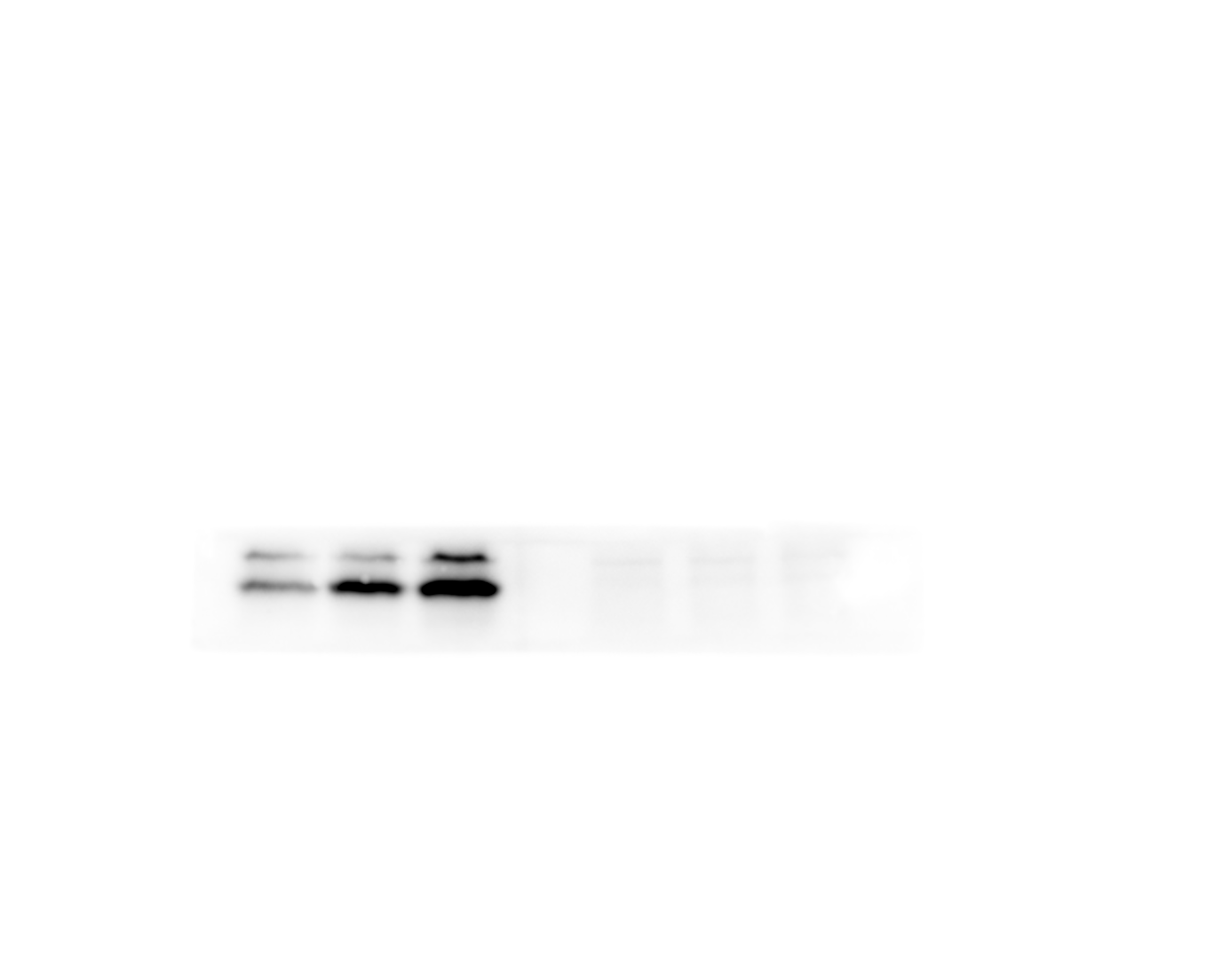


**BCL-2**


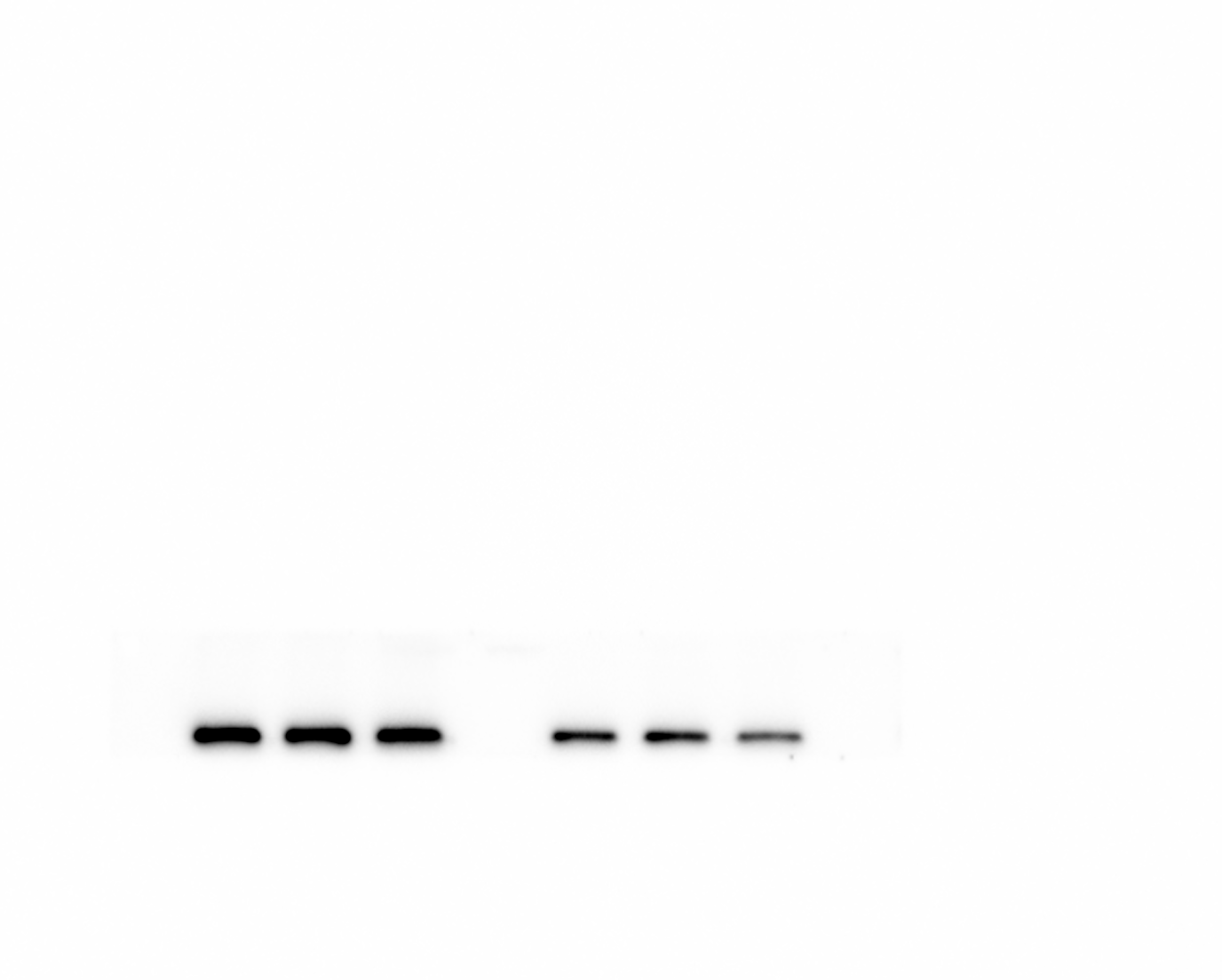


**GAPDH**

**Figure 6 A**


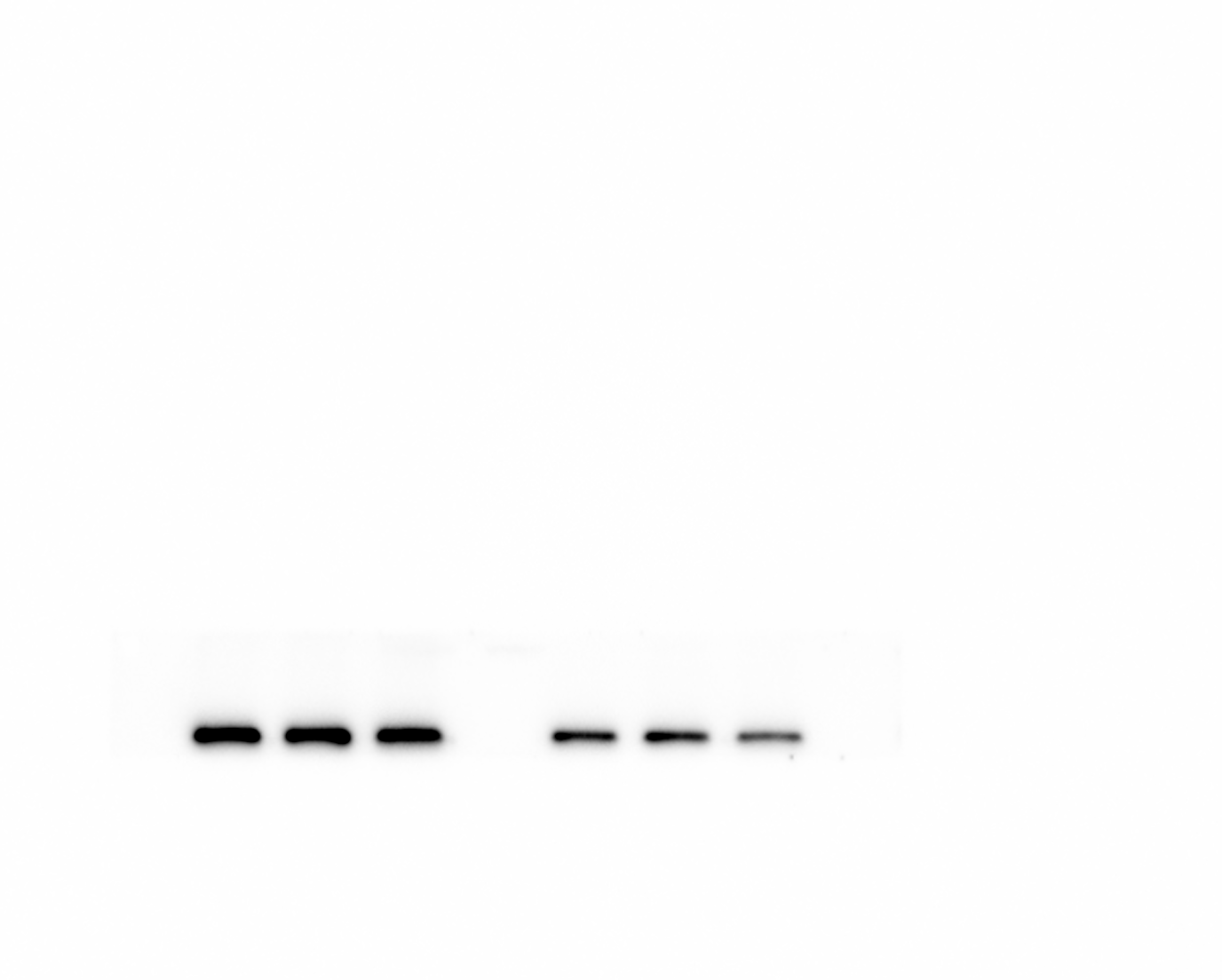


**P38**


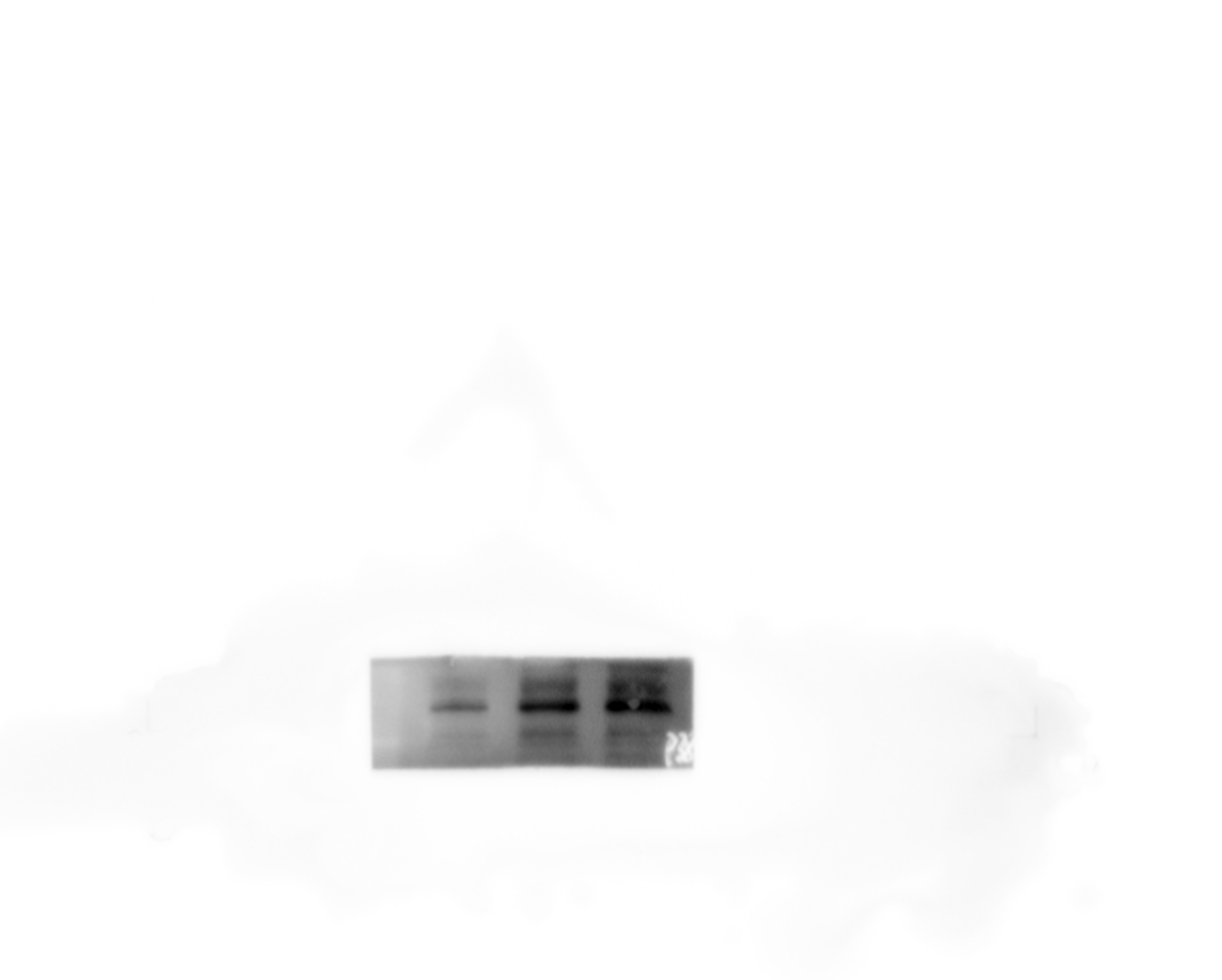


**pP38**


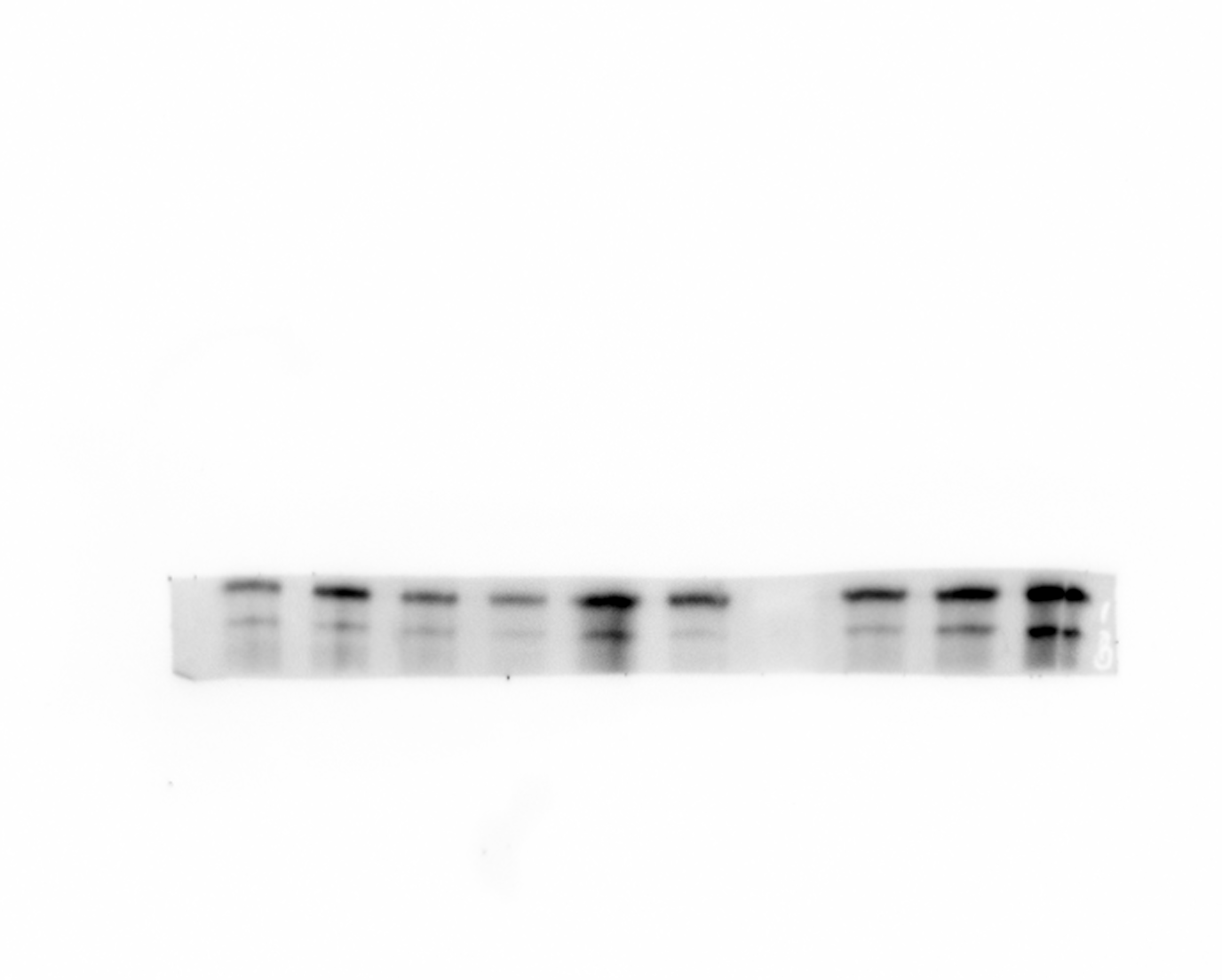


**pP65**


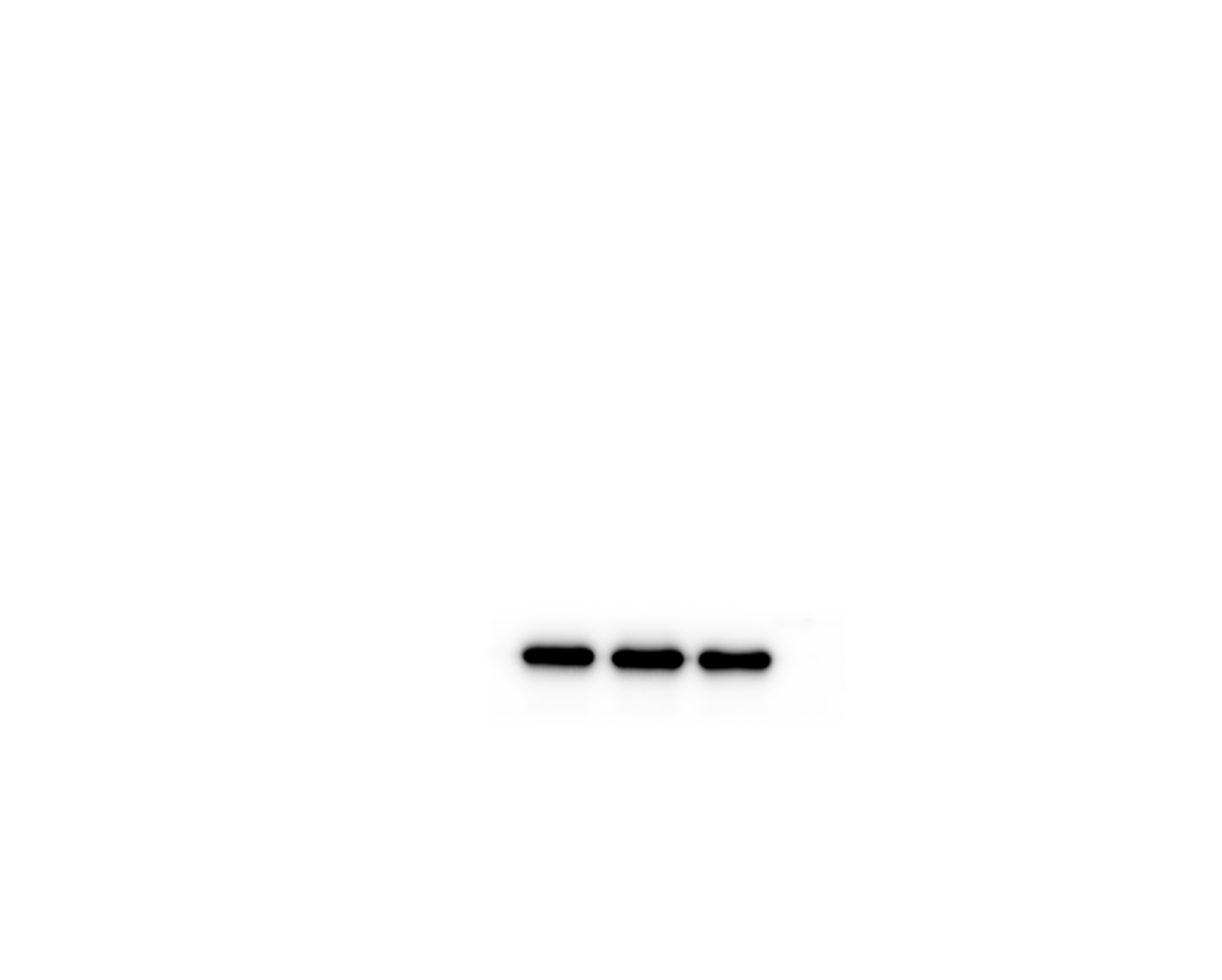


**GAPDH**

**Figure 6 B**


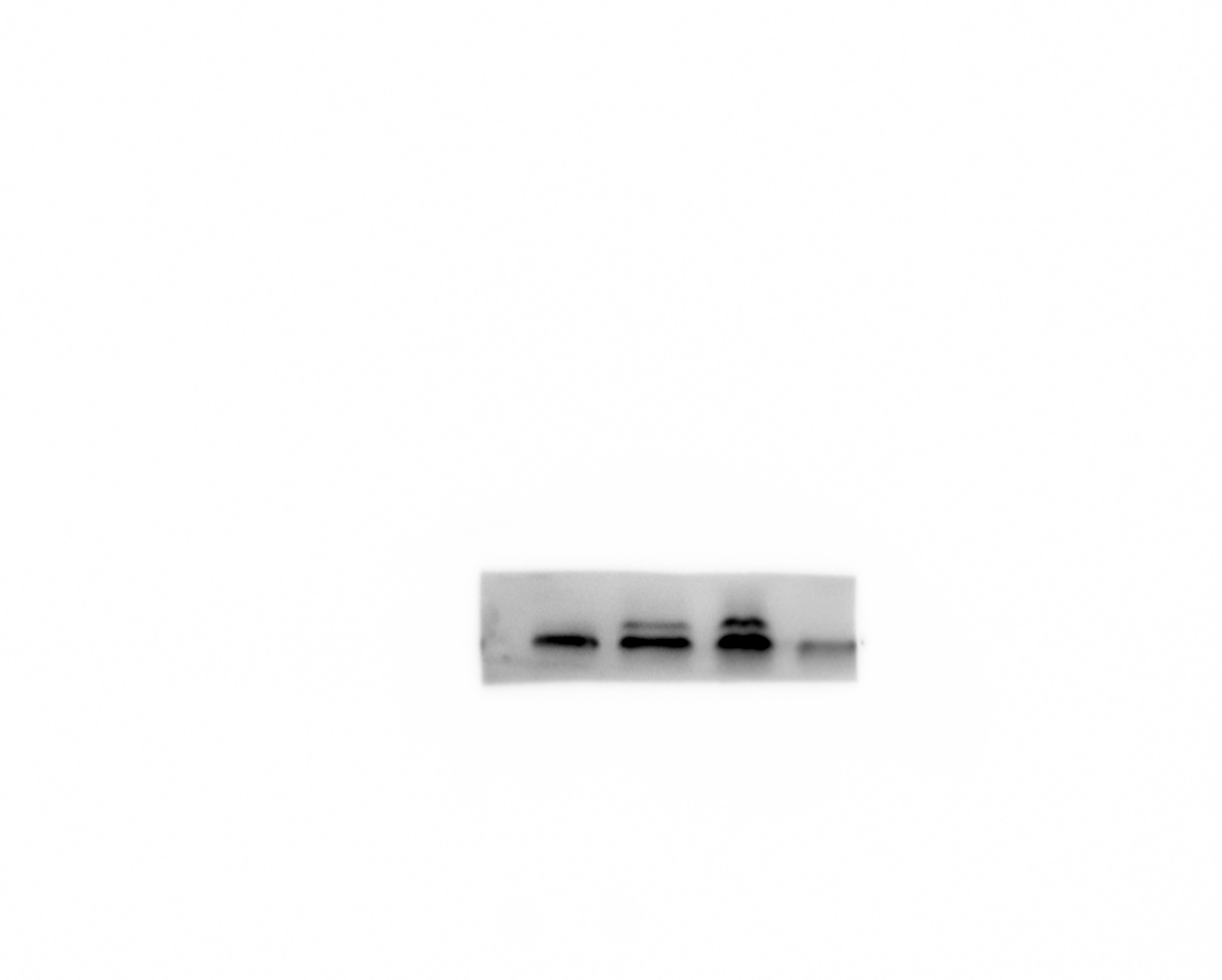


**P38**


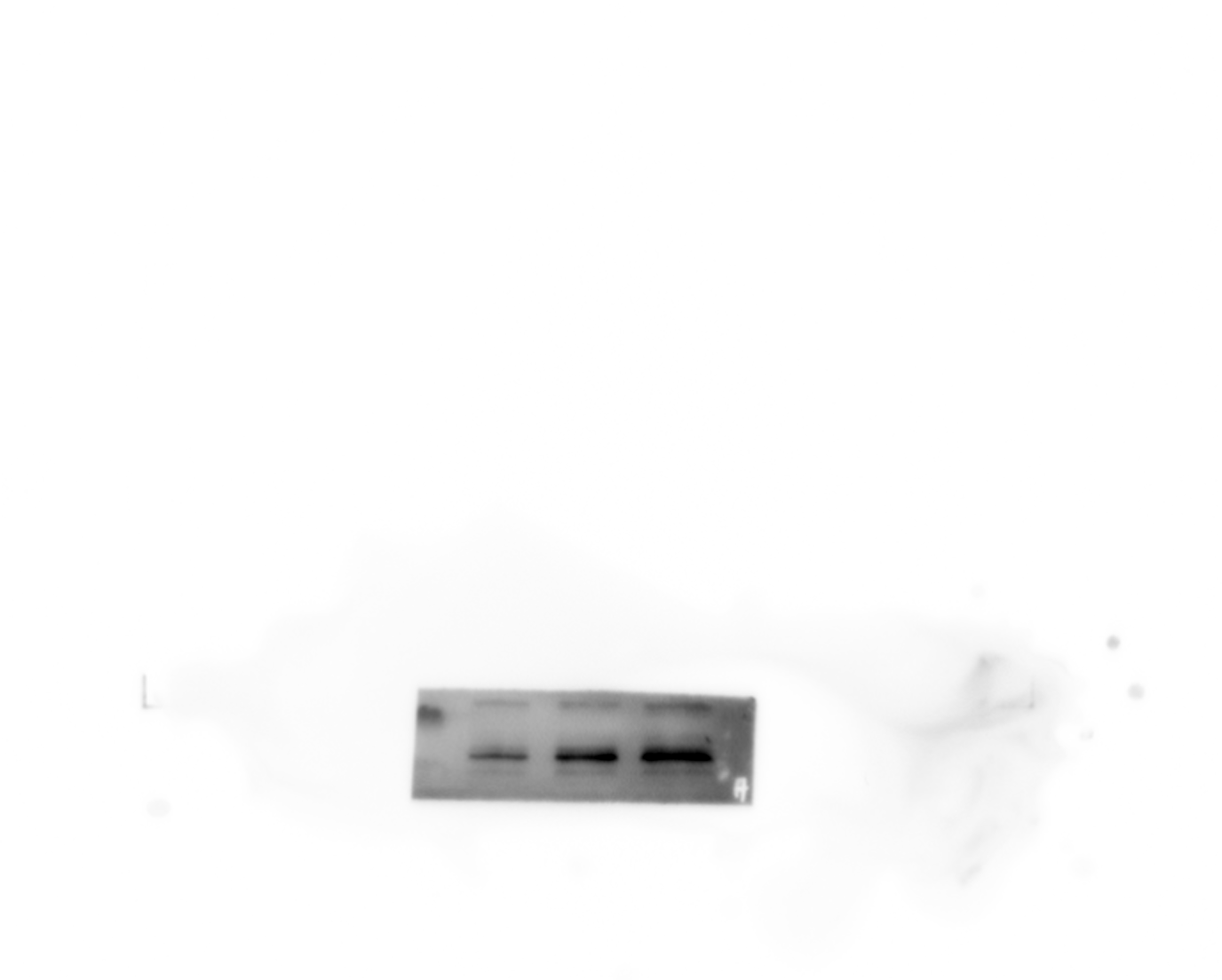


**pP38**


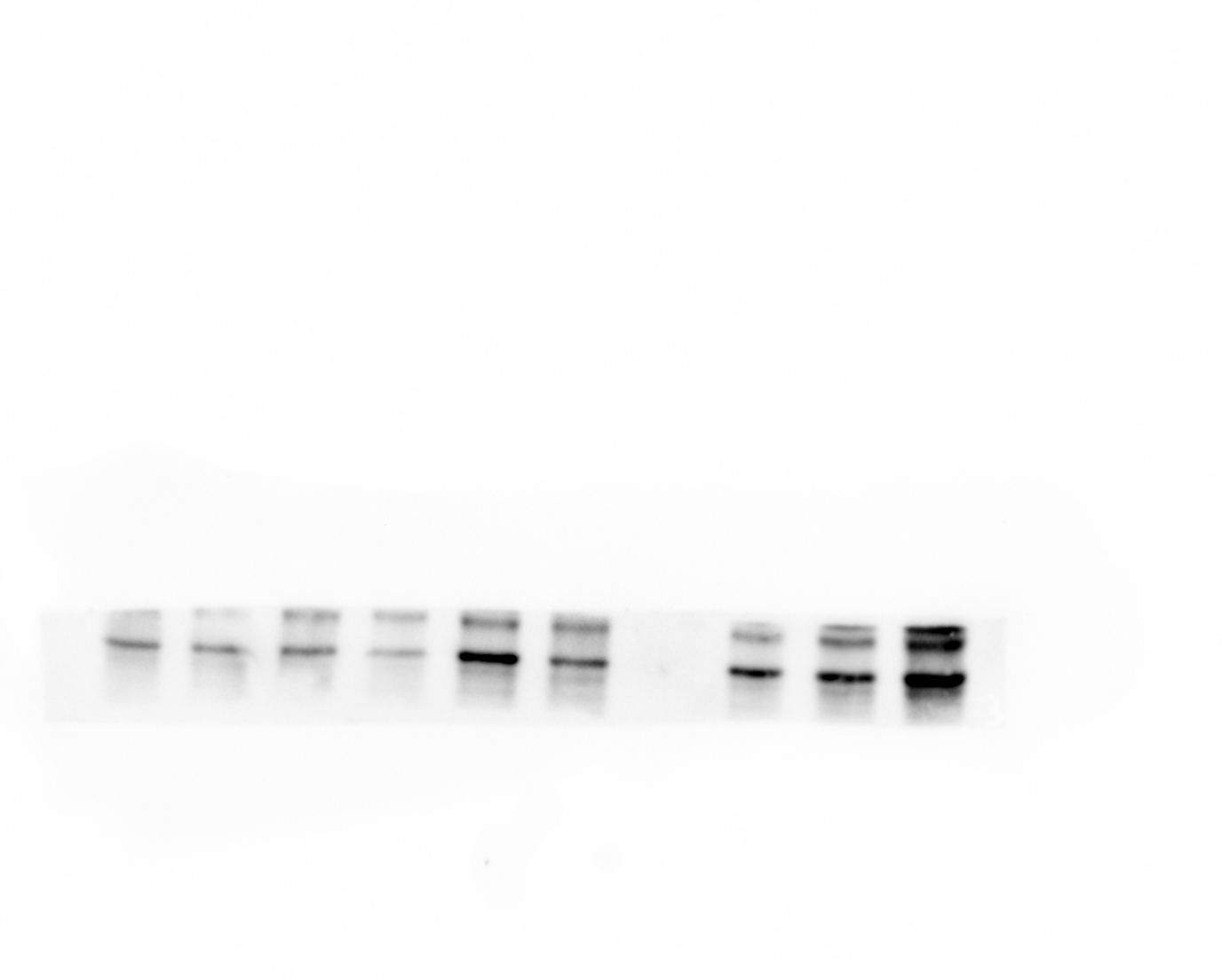


**pP65**


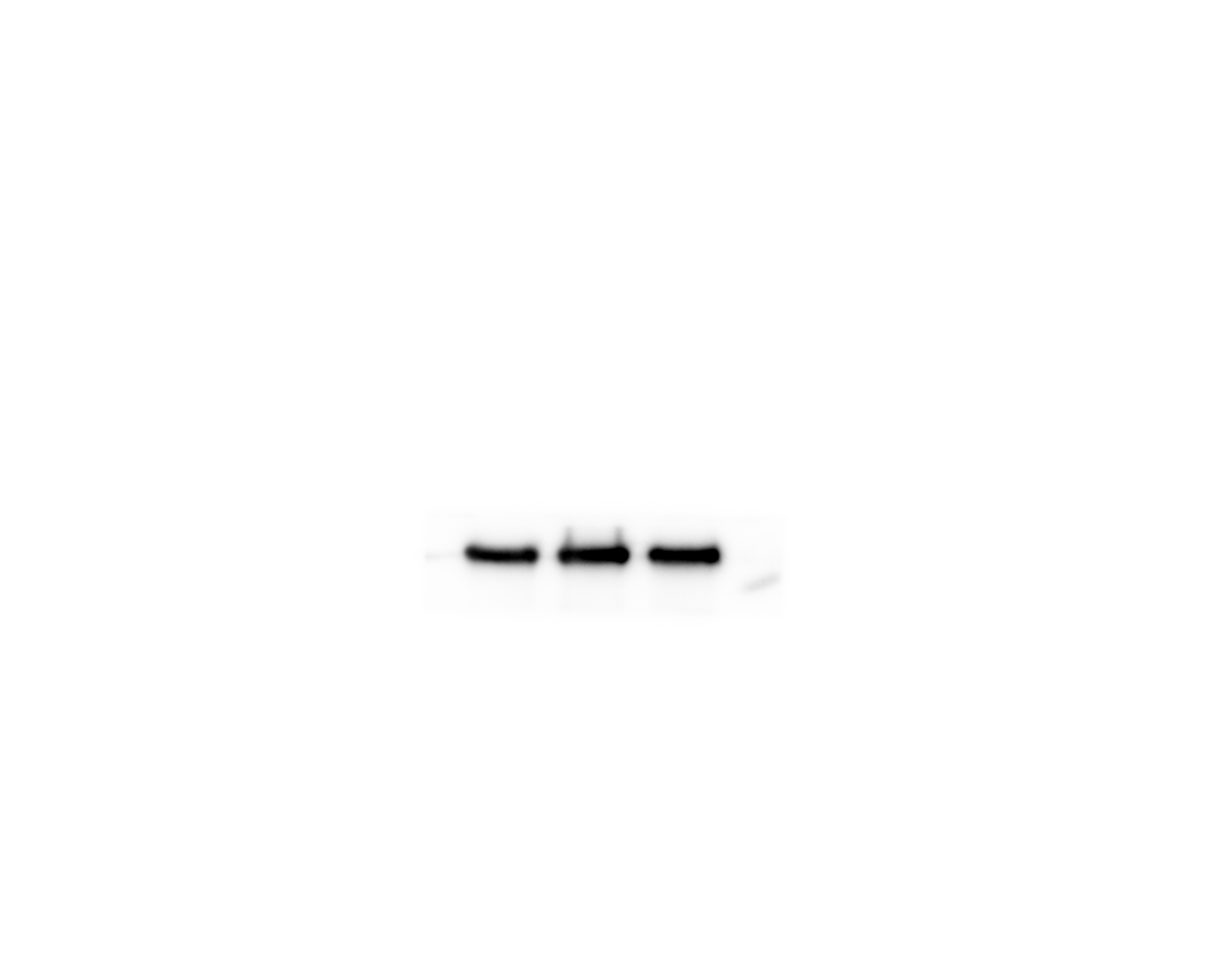


**GAPDH**

**Figure 6 C**


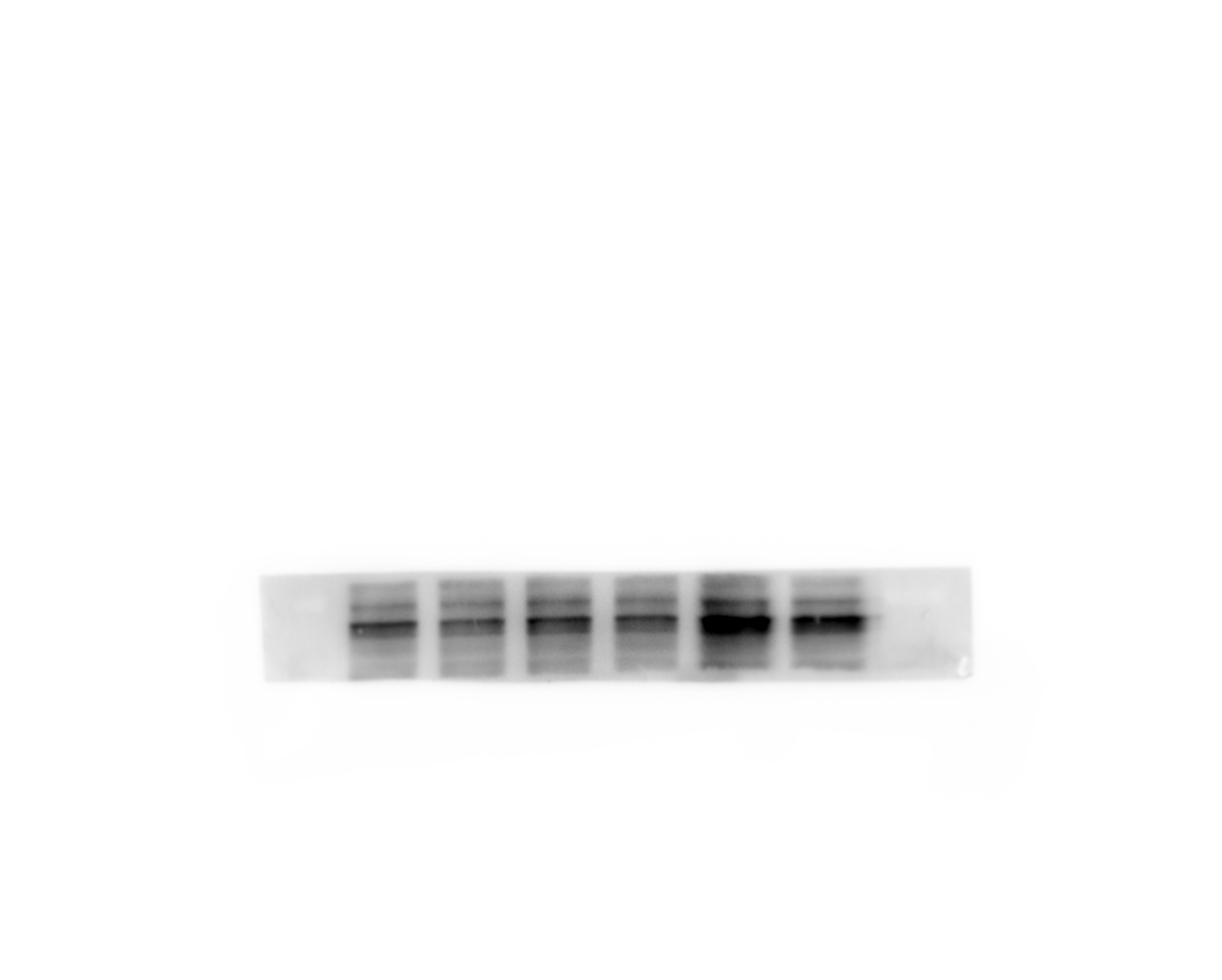


**pP65**


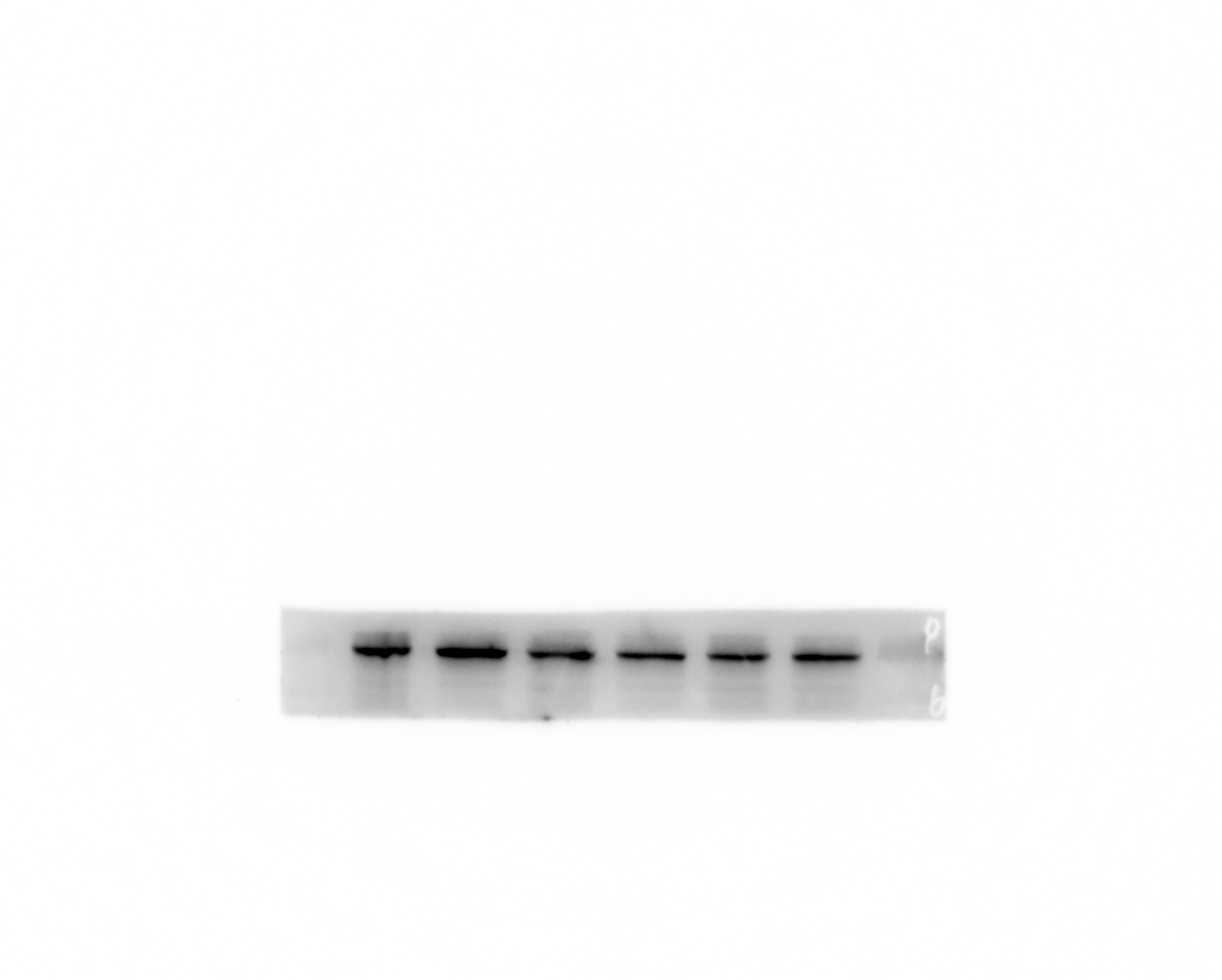


**P38**


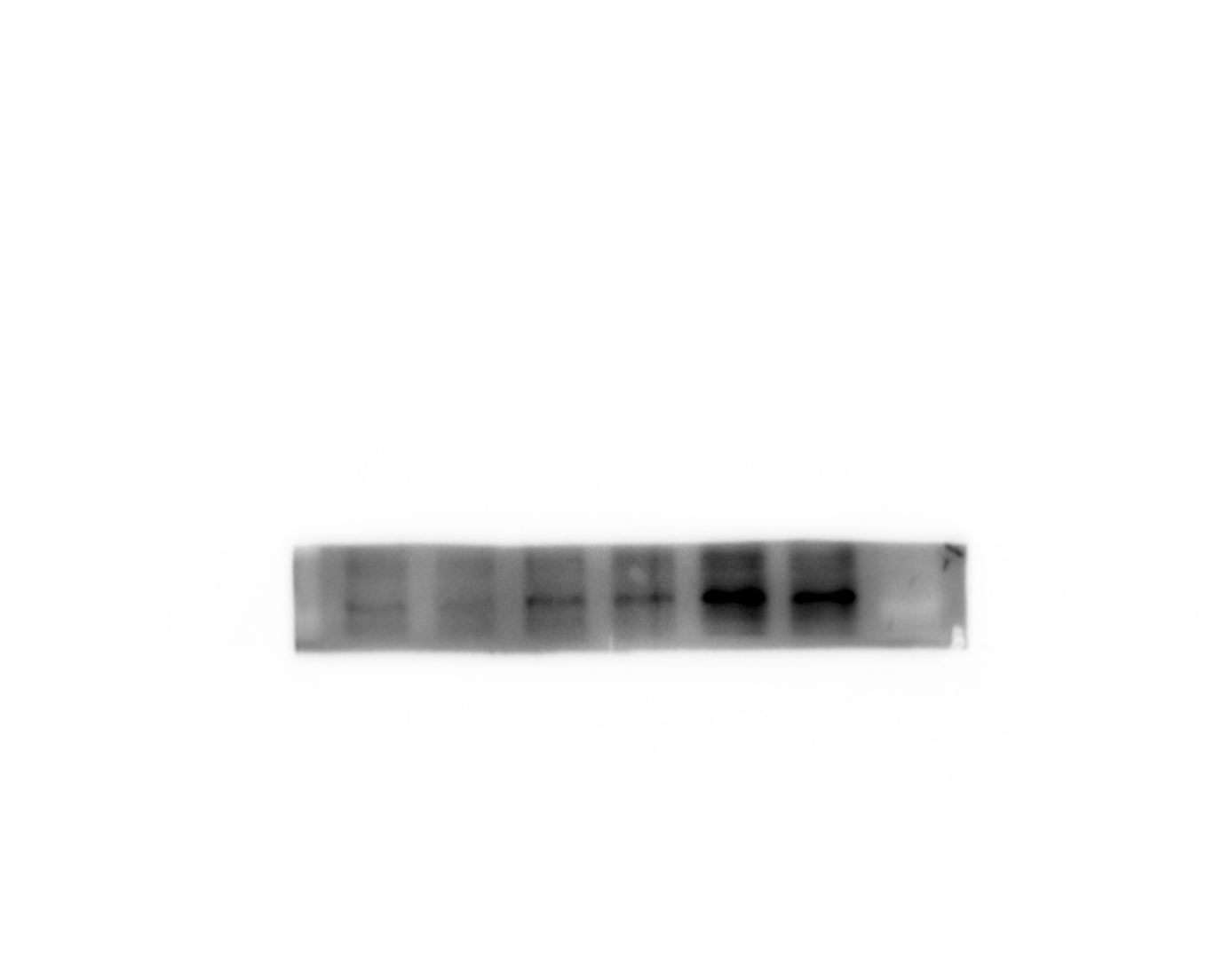


**pP38**


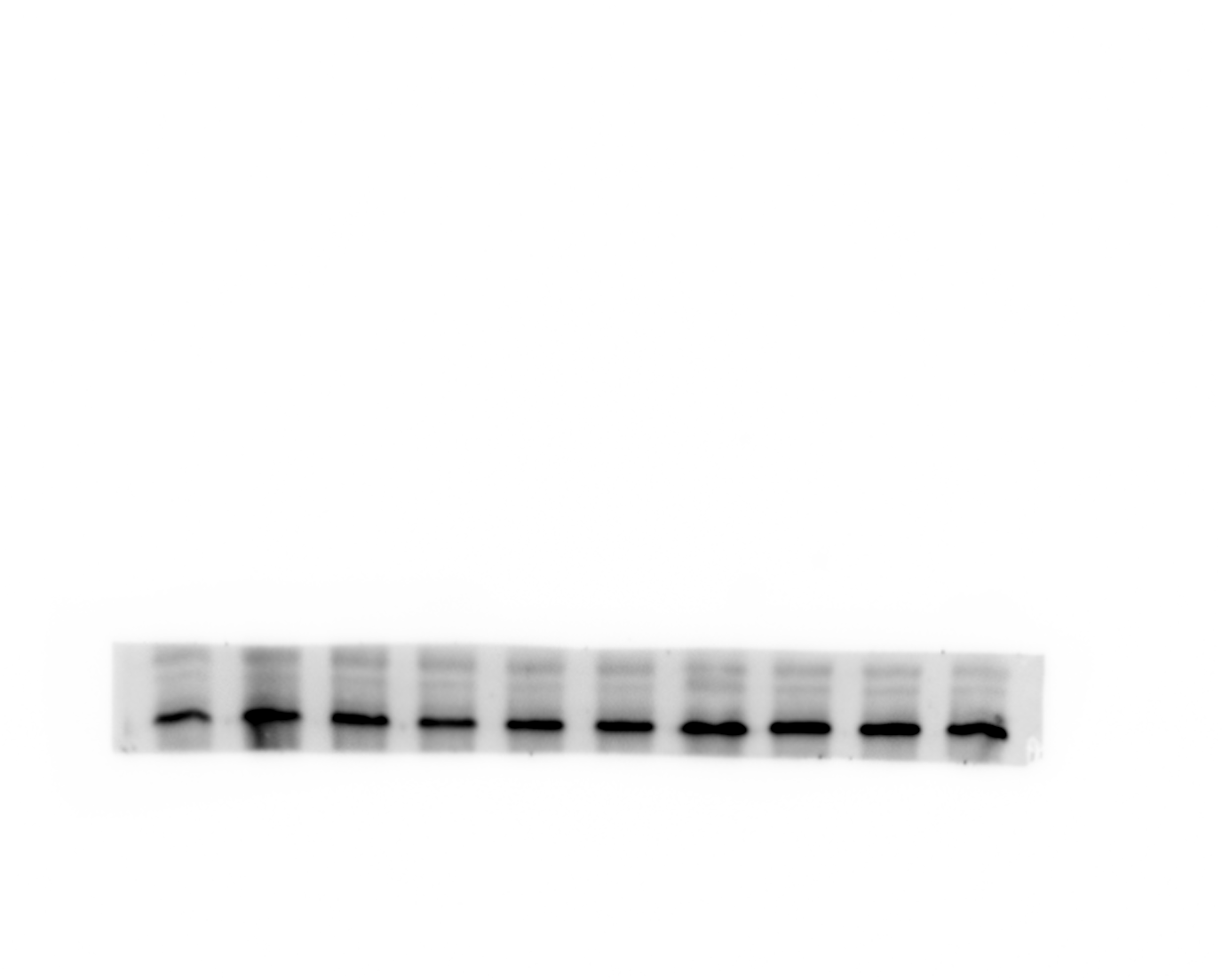


**GAPDH**

**Figure 6 D**


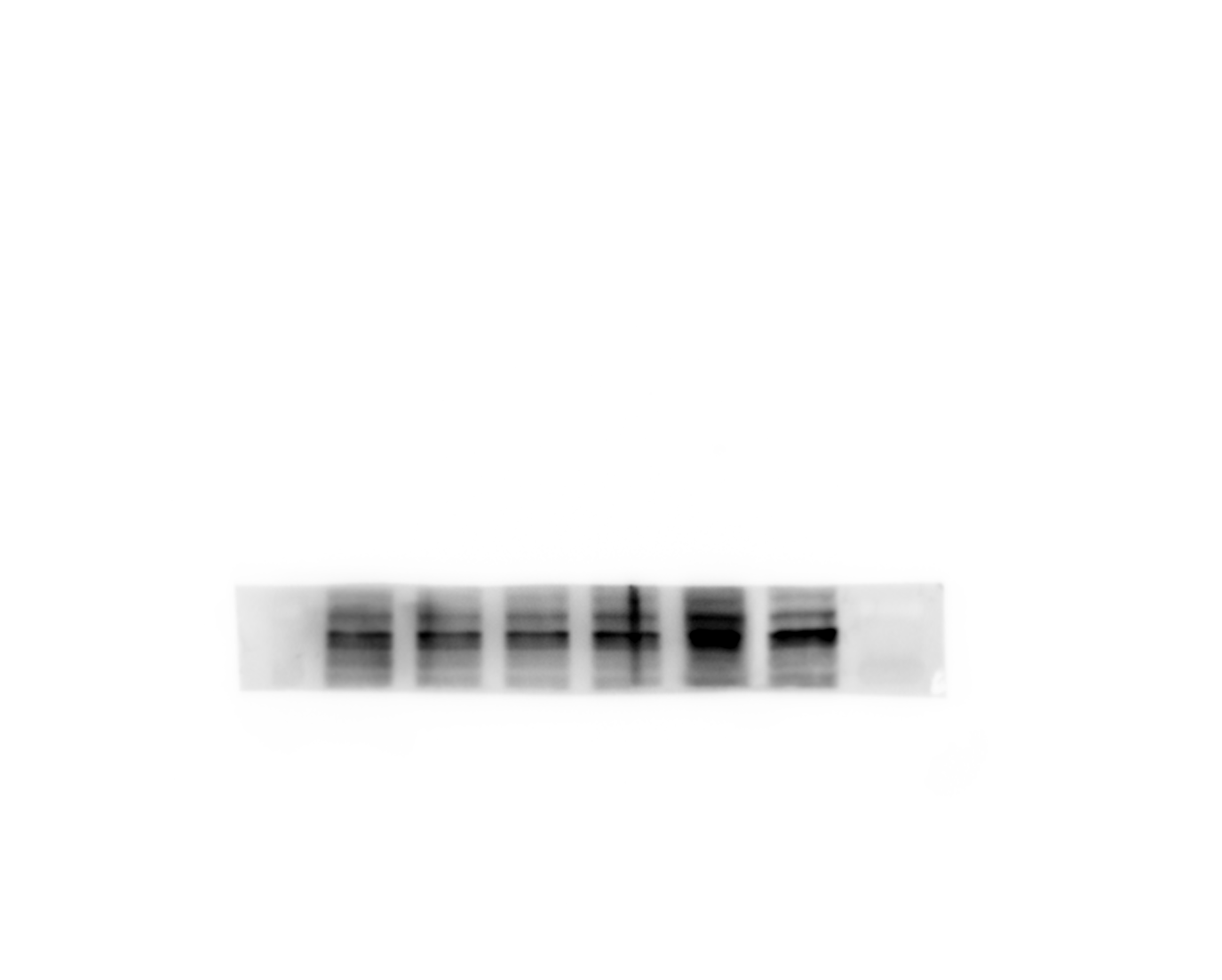


**pP65**


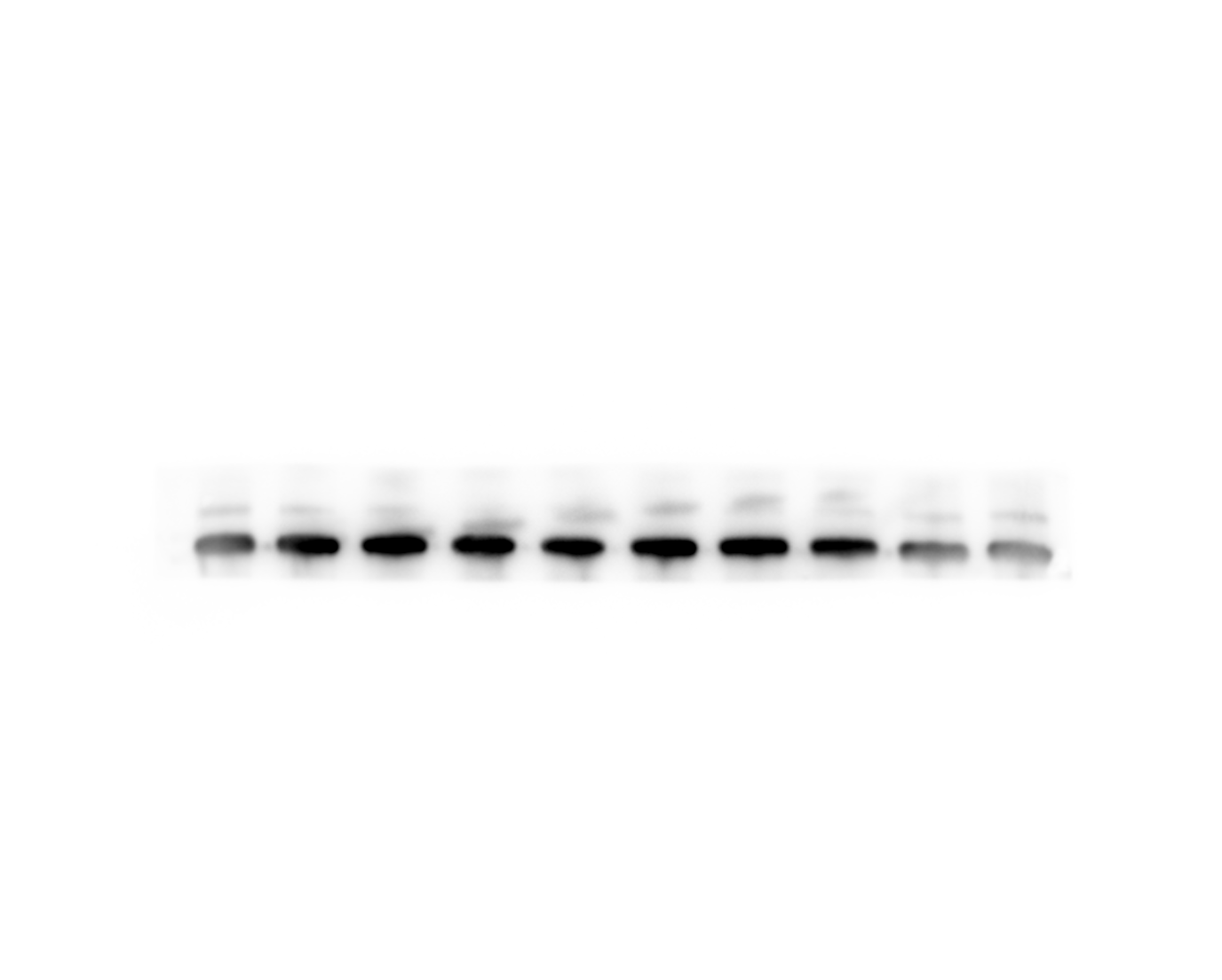

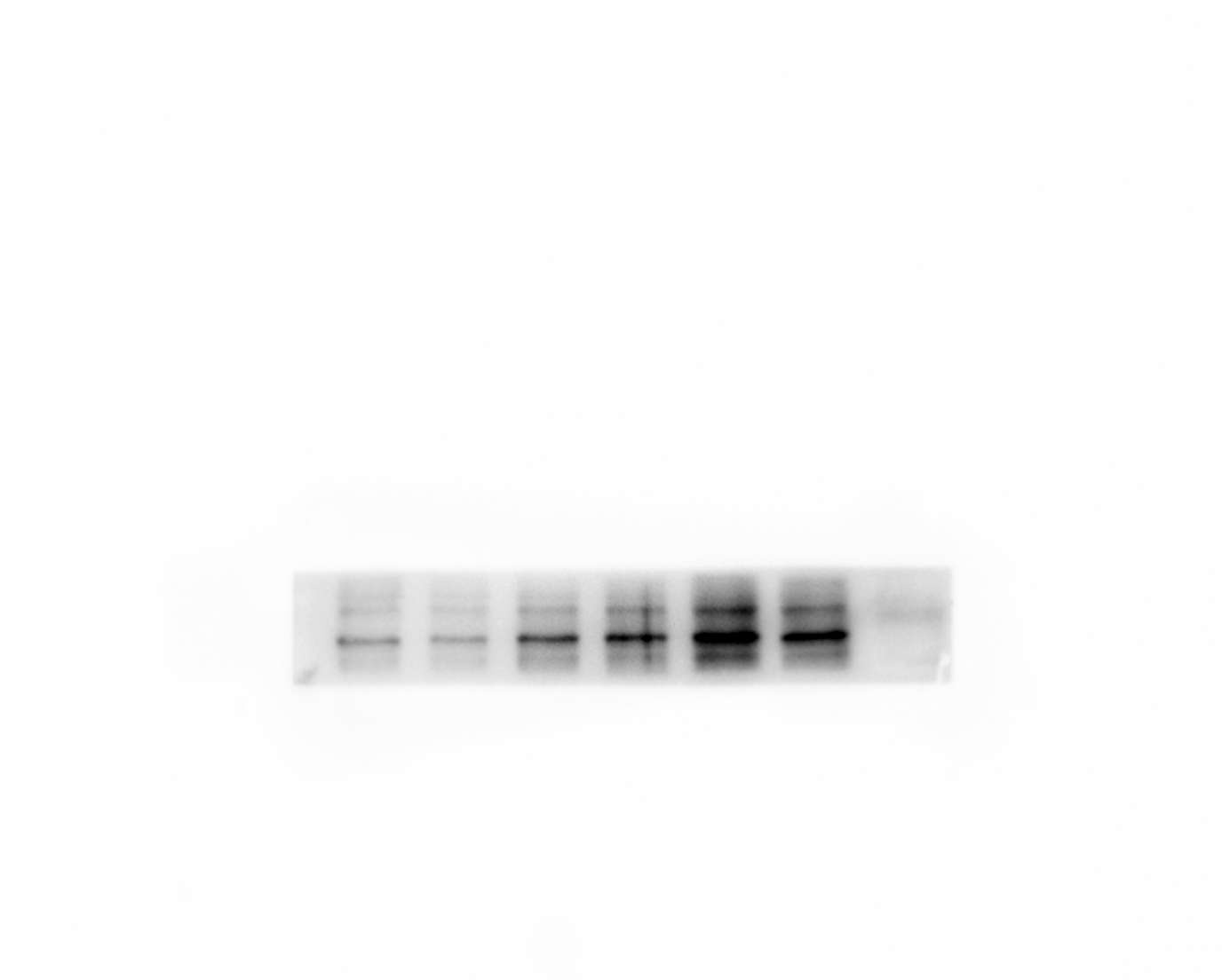

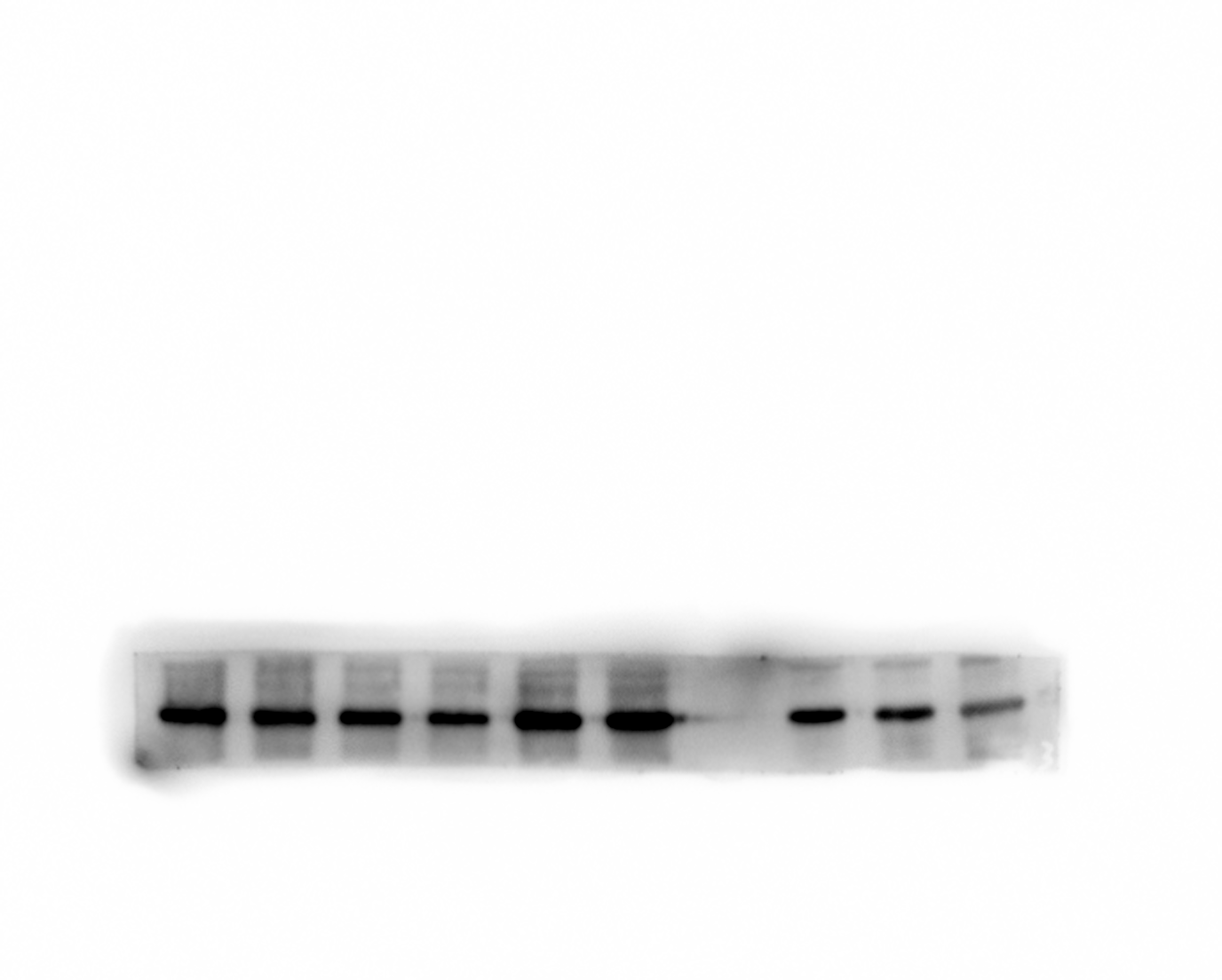


**GAPDH**

**pP38**

**P38**
